# Interplay between chromophore binding and domain assembly by the B_12_-dependent photoreceptor protein, CarH†

**DOI:** 10.1039/d1sc00522g

**Published:** 2021-05-05

**Authors:** Inês S. Camacho, Rachelle Black, Derren J. Heyes, Linus O. Johannissen, Lennart A. I. Ramakers, Bruno Bellina, Perdita E. Barran, Sam Hay, Alex R. Jones

**Affiliations:** Manchester Institute of Biotechnology and Department of Chemistry, The University of Manchester 131 Princess Street Manchester M1 7DN UK perdita.barran@manchester.ac.uk sam.hay@manchester.ac.uk; Photon Science Institute, The University of Manchester Oxford Road Manchester M13 9PL UK

## Abstract

Organisms across the natural world respond to their environment through the action of photoreceptor proteins. The vitamin B_12_-dependent photoreceptor, CarH, is a bacterial transcriptional regulator that controls the biosynthesis of carotenoids to protect against photo-oxidative stress. The binding of B_12_ to CarH monomers in the dark results in the formation of a homo-tetramer that complexes with DNA; B_12_ photochemistry results in tetramer dissociation, releasing DNA for transcription. Although the details of the response of CarH to light are beginning to emerge, the biophysical mechanism of B_12_-binding in the dark and how this drives domain assembly is poorly understood. Here – using a combination of molecular dynamics simulations, native ion mobility mass spectrometry and time-resolved spectroscopy – we reveal a complex picture that varies depending on the availability of B_12_. When B_12_ is in excess, its binding drives structural changes in CarH monomers that result in the formation of head-to-tail dimers. The structural changes that accompany these steps mean that they are rate-limiting. The dimers then rapidly combine to form tetramers. Strikingly, when B_12_ is scarcer, as is likely in nature, tetramers with native-like structures can form without a B_12_ complement to each monomer, with only one apparently required per head-to-tail dimer. We thus show how a bulky chromophore such as B_12_ shapes protein/protein interactions and in turn function, and how a protein can adapt to a sub-optimal availability of resources. This nuanced picture should help guide the engineering of B_12_-dependent photoreceptors as light-activated tools for biomedical applications.

## Abstract 2

The function of the bacterial photoreceptor protein, CarH, is regulated by changes to its oligomeric state. Camacho *et al.* detail how binding of vitamin B_12_ in the dark drives assembly of the protein tetramer that in turn blocks transcription.

## Graphical abstract

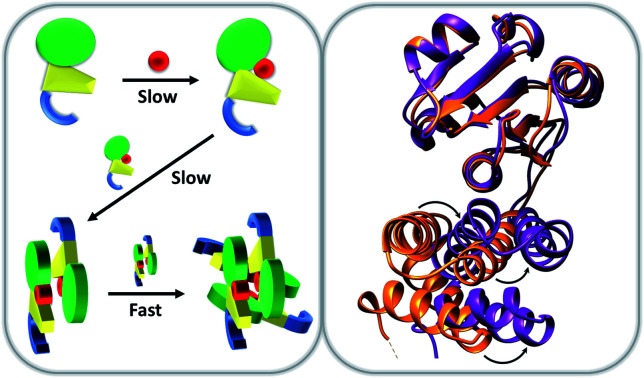

## Introduction

Vitamin B_12_ is the largest and most structurally complex vitamin in nature.^1^ At its center is a highly conjugated cobalamin macrocycle, which enables one active derivative, 5′-deoxyadenosylcobalamin (AdoCbl, Fig. S1a†), to act as a latent source of radicals for numerous mutase^2^ and eliminase^3^ enzymes. This extensive conjugation also means that it absorbs light from across much of the UV and visible regions of the spectrum, making it an ideal chromophore for environmental light-sensing functions in biology. Although the photochemistry of B_12_ species has been studied for decades, its role in photobiology is only now becoming apparent.^4^ The discovery of the bacterial transcriptional regulator, CarH,^5,6^ has brought forth a new area of photobiology based on B_12_ as a photoactive chromophore.^7^ AdoCbl photochemistry in CarH results in transcriptional activation in bacteria, which leads to the biosynthesis of carotenoids in response to photo-oxidative stress. Mechanistic details about its function are now beginning to emerge^4,6–11^ and it is already showing great promise and versatility as the basis of photoactivated, biomolecular tools.^12–16^

The size and structural complexity of AdoCbl reflect a long and expensive biosynthetic pathway^17^ and mean that its uptake^18^ and subsequent binding to its dependent enzymes^19^ are often tightly regulated. The importance of these pathways is highlighted by the genetic disorders that are caused by mutations to the regulatory proteins.^20^ Similarly, the binding of AdoCbl to riboswitches and other proteins is a crucial aspect of its role as a regulator of genetic control elements.^21^ In CarH, this is not only because AdoCbl serves as a photoactive chromophore, but also because its binding triggers the formation of oligomeric forms that bind to, and thus block, operator DNA. The binding of AdoCbl to CarH from *Thermus thermophilus* (TtCarH), for example, converts apo-monomers into holo-tetramers that bind DNA.^6^ Light absorption by AdoCbl then results in the disassembly of this protein/DNA complex and transcriptional activation. We now have some insight into the structural^10^ and mechanistic^11,22^ basis of the light-dependent activation. By contrast, to date there has been no biophysical investigation into how the binding of AdoCbl drives the assembly of CarH oligomers. This not only limits our understanding of how it achieves transcriptional regulation in nature but also holds back tool development and optimization.

Here, we have probed the binding of AdoCbl to TtCarH and subsequent oligomer assembly using native ion mobility mass spectrometry, time-resolved spectroscopy and molecular dynamics (MD) simulations. This powerful combination of biophysical techniques has afforded new insights that detail the dynamic interplay between the binding of the chromophore and subunit assembly.

## Results

### AdoCbl binding drives the structural changes in TtCarH that facilitate oligomerization

**Fig. 1:**
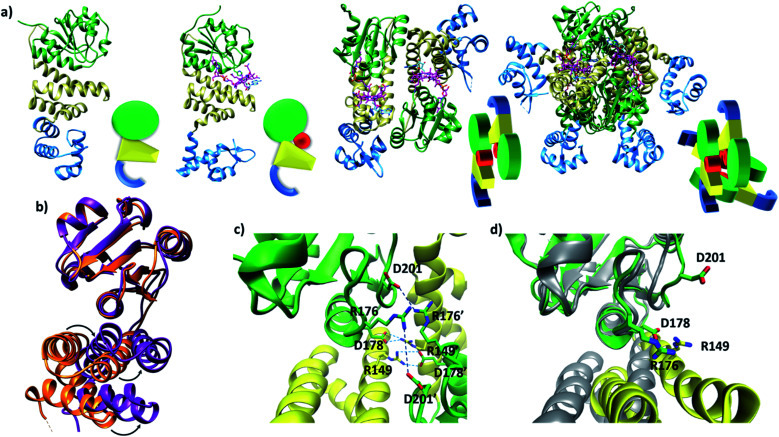
(a) TtCarH structures and their cartoon representations, colored by their domain: B_12_-binding (green), 4-helix bundle (yellow), DNA-binding (blue), and AdoCbl (magenta). L–R: apo-monomer (representative from MD simulations), holo-monomer, holo-dimer, and holo-tetramer (all PDB: 5C8D). (b) 4-helix bundle (bottom) positions in each monomer aligned to the B_12_-binding domain (top): holo-TtCarH (orange, PDB: 5C8D) and apo-TtCarH (purple, simulated). A different color scheme to panel (a) is used to distinguish between different structures. Arrows indicate helical displacement in apo-TtCarH relative to holo-TtCarH (see also Fig. S3a and b†). (c) Close-up of the interface between monomer units in the holo-TtCarH head-to-tail dimers with salt-bridges identified here (D178–R149) and previously^10^ (D201–R176) highlighted. (d) The same interfacial region illustrated in panel (c), but now of the simulated apo-TtCarH monomer. The change in 4-helix bundle conformation relative to holo-TtCarH (in grey) results in a helix moving into the dimer interface such that the key salt-bridge residues are no longer able to stabilize the head-to-tail dimers (see also Fig. S3c†).

Published structural data have revealed much about the tertiary and quaternary structure of TtCarH.^10^ Each monomer comprises a N-terminal DNA-binding domain, a 4-helix bundle and a Rossman fold (B_12_-binding) domain at the C-terminus (Fig. 1a). Although the quaternary structure is formally a homo-tetramer when AdoCbl is bound, it is perhaps better described as a dimer of head-to-tail dimers (Fig. 1a). AdoCbl binds to the Rossman fold in a conformation where the lower axial 5,6-dimethylbenzimidazole base is displaced and the Co is instead coordinated by H177 (Fig. S1b†).^6,10^ The position of AdoCbl between the Rossman fold and the 4-helix bundle places the upper axial 5′-deoxyadenosyl ligand (Ado) in steric contact with W131 from the 4-helix bundle (Fig. S1b†).^10^ This is thought to force each holo-TtCarH monomer to adopt an upright conformation that facilitates the formation of head-to-tail dimers. In the absence of structural data for apo-TtCarH, however, this hypothesis has not been confirmed.

The lack of structural data for apo-TtCarH is presumably because it is prone to precipitation at higher concentrations. We have therefore performed MD simulations (see the Experimental section and Fig. S2†) of apo-TtCarH, which confirm the above hypothesis. From what is known about the photoconversion mechanism,^10,11^ when Ado is photo-dissociated, W131 moves into the vacated space, resulting in a displacement of the 4-helix bundle (Fig. S3a†) and the formation of a *bis*-histidine adduct with H132 (Fig. S1b†).^10,11^ It appears that the movement of the 4-helix bundle then disrupts the head-to-tail dimer interface, thus driving tetramer dissociation. Our MD simulations of apo-TtCarH confirm that in the absence of the entire AdoCbl the 4-helix bundle is displaced relative to the dark structure (8.14 ± 1.33 Å) akin to when Ado is photo-dissociated (9.7 Å), but to a slightly different position (Fig. 1b, S3a and b†).

It might seem surprising that such a significant structural change occurs on the relatively short timescale of these MD simulations (400 ns). This would be a reasonable concern if the simulations started from a near-equilibrium structure, which is not the case here. Briefly, the crystal structure of photo-converted holo-TtCarH (*i.e.*, with the Ado missing; PDB: 5C8F)^10^ was taken and the rest of the cobalamin was then removed *in silico*. Three simulations were run in parallel (Fig. S2b†) after energy minimization. Consistent with the mechanism described above, we have demonstrated in previous MD simulations^11^ that removing just the Ado from holo-TtCarH *in silico* causes a rapid conformational change due to the steric strain of the protein pushing against the Ado. It is therefore reasonable that removing the entire cobalamin triggers a conformational change towards a new equilibrium conformation on the simulation timescale.

In the published structure of holo-TtCarH,^10^ two charged residues, R176 and D201, were identified at the surface of each monomer unit of holo-TtCarH, which form salt-bridges that stabilize the head-to-tail dimers. We have identified two further residues, R149 and D178, which could feasibly fulfill a similar role (Fig. 1c). In the photo-converted state these residues are no longer able to readily form salt-bridges. Our MD simulation structures suggest a similar situation for apo-TtCarH relative to the holo-TtCarH structure (Fig. 1d and S3c†). Our simulations therefore provide strong additional evidence in support of the hypothesis that the binding of AdoCbl to TtCarH triggers the structural change that facilitates oligomer formation.

### For TtCarH tetramers to form, it is not necessary for AdoCbl to be bound to each monomer

**Fig. 2:**
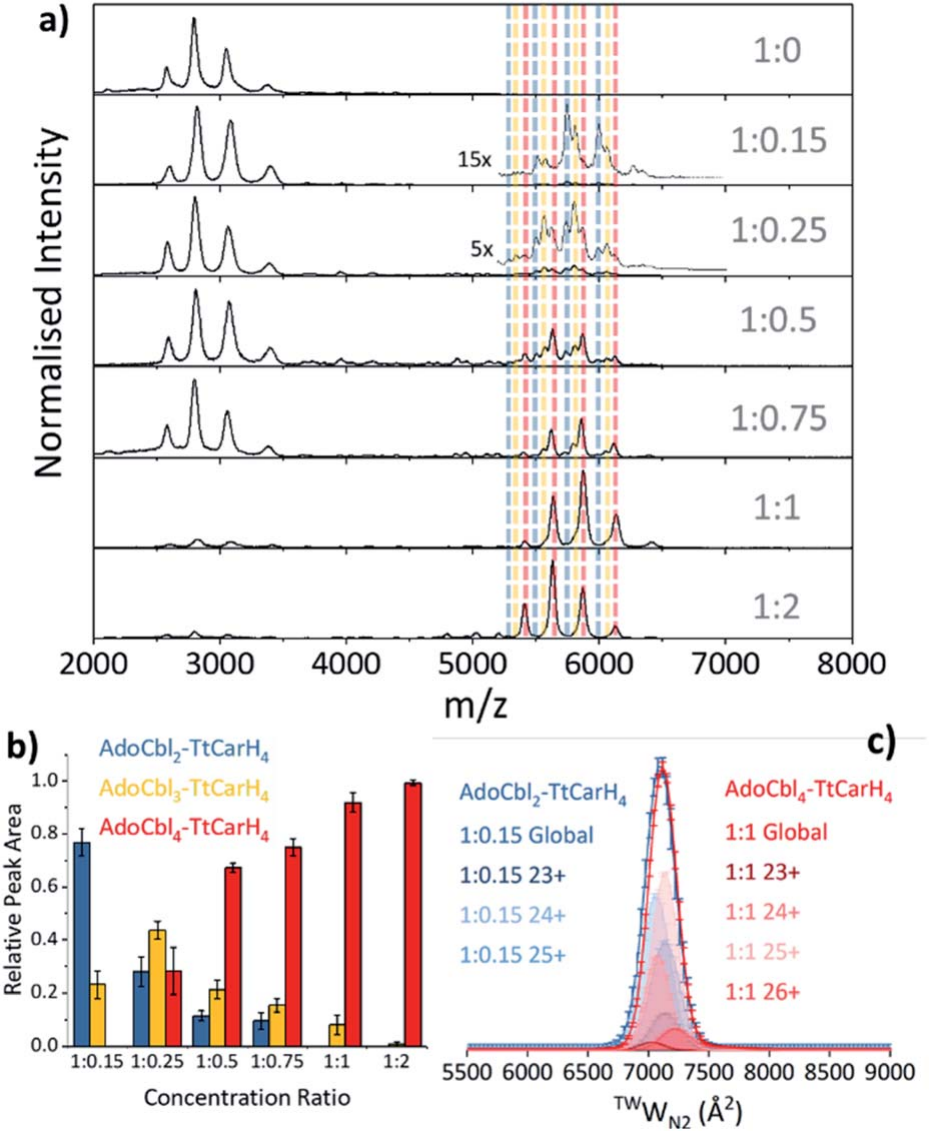
(a) Native mass spectra of WT TtCarH with increasing (top to bottom) molar ratios of AdoCbl. Vertical dashed lines highlight peaks from different tetramer species and are color-coded as indicated in panel (b). (b) Relative peak area as a function of WT TtCarH : AdoCbl ratio for the mass spectral signals highlighted in panel (a), which correspond to different WT TtCarH tetramer species. (c) ^TW^CCS_N2_ distributions from ion mobility data for the major tetramer species present at both 1 : 0.15 (blue, AdoCbl_2_–TtCarH_4_) and 1 : 1 (red, AdoCbl_4_–TtCarH_4_). Each global ^TW^CCS_N2_ is the summation of the various charge states, corrected for their peak area and height.

Our native ion mobility mass spectrometry data indicate that, although AdoCbl-binding drives tetramer formation, each tetramer does not need to comprise four holo-monomers. Data were acquired for wild-type (WT) TtCarH samples (10 μM apo-protein) containing AdoCbl ranging from sub-stoichiometric quantities to a two-fold excess (Fig. 2a). In the absence of AdoCbl, the spectrum is dominated by signals from the apo-monomer with a low population of apo-dimer, both presenting over narrow charge state distributions. Ion mobility data show little variance in the collision cross sections (^TW^CCS_N2_) across the monomer charge states, implying that they are compact and homogeneous forms (Fig. S4a†). With increasing concentrations of AdoCbl, the apo-monomer is gradually replaced by signals predominantly from tetrameric species (Fig. 2a). The holo-tetramer with a full complement of AdoCbl has a mass of ∼140.5 kDa. Strikingly, at lower TtCarH : AdoCbl ratios, tetramer populations are observed with sub-stoichiometric AdoCbl, *i.e.*, two (∼137.3 kDa) or three (∼138.9 kDa) AdoCbl per tetramer (Fig. 2b). Each form presents with a charge state distribution similar to the tetramer with four AdoCbl bound (*i.e.*, 23 to 26+; Fig. 2c, S4 and S5†). This observation indicates that, when the B_12_ chromophore is scarce, tetramers form without a full complement of AdoCbl.

For the sake of clarity, we will now use a systematic nomenclature to define different TtCarH oligomers with different B_12_ stoichiometries. Protein with no B_12_ bound will be referred to as apo-TtCarH_*x*_, where *x* signifies the oligomeric state (*e.g.*, apo-TtCarH_2_ for dimers). For the holo-protein we will also indicate the type and number of B_12_ species bound: B12_*y*_–TtCarH_*x*_ (*e.g.*, AdoCbl_2_–TtCarH_4_ for tetramers with two AdoCbl bound).

As the concentration of AdoCbl is increased, AdoCbl_4_–TtCarH_4_ (∼140.5 kDa) dominates, suggesting that this is the thermodynamically favored form. Perhaps surprisingly, the narrow, invariant charge state distributions (Fig. 2a) and ion mobility data (Fig. 2c, S4 and S5†) suggest that there is little conformational variation between AdoCbl_2_–TtCarH_4_, AdoCbl_3_–TtCarH_4_ and AdoCbl_4_–TtCarH_4_. Each has a comparable ^TW^CCS_N2_ distribution, consistent with all tetrameric forms adopting a similar quaternary arrangement. This fact, along with the absence of a signal from AdoCbl_1_–TtCarH_4_, suggests that one AdoCbl_1_–TtCarH_1_ is enough to provide a structural ‘template’ for a partnering apo-TtCarH_1_ to adopt the correct conformation in each head-to-tail dimer for tetramers to form.

It is also possible that AdoCbl binds directly to apo-TtCarH_2_. The low intensity dimer signals in Fig. 2a are from apo-TtCarH_2_, AdoCbl_1_–TtCarH_2_ and AdoCbl_2_–TtCarH_2_ (Fig. S6†). Moreover, there is only the slightest suggestion of a signal from AdoCbl_1_–TtCarH_1_, and only when AdoCbl is in a two-fold excess (Fig. S7†). This last observation is consistent with either preferential binding to apo-TtCarH_2_ or the fact that AdoCbl binding to the apo-TtCarH_1_ simply drives the position of equilibrium overwhelmingly towards AdoCbl_2_–TtCarH_2_. Either way, the low population of dimers suggests that when holo-dimers do form, they rapidly combine to form tetrameric species. This is supported by the fact that the relative populations of the dimer species follow a different pattern with increasing AdoCbl concentration to the various tetramer species (Fig. S6†). Here, apo-TtCarH_2_ remains a significant sub-population, including when AdoCbl is in excess.

### B_12_ binds to both monomeric and dimeric TtCarH

**Fig. 3:**
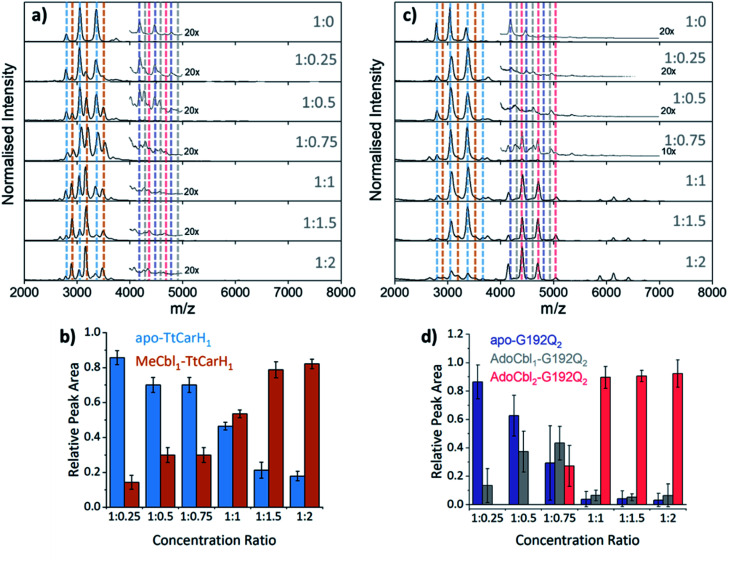
(a) Native mass spectra of WT TtCarH with increasing (top to bottom) molar ratios of MeCbl. Vertical dashed lines highlight peaks from different monomer and dimer species, which are color-coded as indicated in panel (b) and S9,† respectively. (b) Relative peak area as a function of WT TtCarH : MeCbl ratio for the mass spectral signals highlighted in panel (a), which correspond to different WT TtCarH monomer species. (c) Native mass spectra of G192Q with increasing (top to bottom) molar ratios of AdoCbl. Vertical dashed lines highlight peaks from different monomer and dimer species, which are color-coded as indicated in S10† and panel (d), respectively. (d) Relative peak area as a function of G192Q : AdoCbl ratio for the mass spectral signals highlighted in panel (c), which correspond to different G192Q dimer species.

To probe the role of dimer intermediates further, we investigated the binding of methylcobalamin (MeCbl), again using native ion mobility mass spectrometry with samples containing the same range of TtCarH : B_12_ ratios. Although MeCbl is known to bind to TtCarH, size exclusion chromatography (SEC) and isothermal calorimetry suggest that the protein remains in the monomeric form.^6^ This is almost certainly because MeCbl lacks both the steric bulk of AdoCbl in the upper axial position (Fig. S1a and S8†) to cause the structural changes that drive TtCarH oligomerization and the capacity to form any necessary stabilizing interactions that are present between the Ado group and the protein. As before, in the absence of MeCbl the mass spectrum of TtCarH contains signals mainly from apo-TtCarH_1_, with a low population of apo-TtCarH_2_ (Fig. 3a). As the MeCbl concentration increases, the apo-TtCarH_1_ population is displaced by signals from MeCbl_1_–TtCarH_1_ (Fig. 3a and b) and apo-TtCarH_2_ evolves to MeCbl_1_–TtCarH_2_ and MeCbl_2_–TtCarH_2_ (Fig. 3a and S9†). These data confirm that B_12_ species can bind directly to both apo-TtCarH_1_ and apo-TtCarH_2_. Across the range of ratios, however, the overall dimer populations remain very low. The implications of this are two-fold. First, direct binding to apo-TtCarH_2_ is a subsidiary as opposed to the preferential route. Second, unlike for AdoCbl, MeCbl binding to apo-TtCarH_1_ does not shift the position of equilibrium towards the dimeric form. The fact that MeCbl-bound dimers have a more significant population relative to apo-TtCarH_2_ than the equivalent AdoCbl-bound species is consistent with their populations not being depleted to form tetramers.

We also investigated the binding of the native chromophore, AdoCbl, to a TtCarH variant that predominantly forms dimers rather than tetramers. G192Q places bulky glutamine residues at the interface between head-to-tail dimers, which sterically hinder the formation of tetramers following chromophore binding.^10^ The mass spectra (Fig. 3c) reveal that the apo-G192Q_1_ (main population) and apo-G192Q_2_ (minor population) signals are replaced upon increased AdoCbl concentration with signals not only from AdoCbl_2_–G192Q_2_ as expected (the dominant species at a 1 : 1 ratio or in excess) but also from AdoCbl_1_–G192Q_1_ and AdoCbl_1_–G192Q_2_. Again, these data are consistent with B_12_ species binding directly to both apo-TtCarH_1_ and apo-TtCarH_2_. In a similar way to the WT tetramers with fewer than four AdoCbl bound (Fig. 2a), AdoCbl_1_–G192Q_2_ makes up a significant population of dimers when AdoCbl is at low concentration (Fig. 3d). There is a more significant sub-population of AdoCbl_1_–G192Q_1_ (Fig. S10a†) when compared to WT (Fig. S7†), which becomes increasingly apparent at higher AdoCbl concentrations. This indicates a dynamic equilibrium between oligomeric states, which is highly dependent both on AdoCbl concentration and on the stability of the tetramer. This would explain why there is little evidence of AdoCbl_1_–TtCarH_1_ in the WT protein – where the tetramer is presumably highly stable – compared to the G192Q ‘dimer’ variant. Tetramers with a slightly smaller ^TW^CCS_N2_ than the WT protein are observed for G192Q, suggesting a stable, compact form, but only when AdoCbl is around stoichiometric concentrations relative to G192Q, or in excess (Fig. 3c, S10b and S11b†). They are therefore likely to be non-specific in their formation and only form when AdoCbl_2_–G192Q_2_ is the dominant species.

Taken together, our mass spectrometry data reveal that TtCarH tetramers can form with fewer than four AdoCbl bound, but that at least one AdoCbl appears to be required per head-to-tail dimer. The ion mobility data suggest that these forms adopt structures similar to AdoCbl_4_–TtCarH_4_. They are also in significant populations when AdoCbl is at sub-stoichiometric concentrations relative to the protein, which is not an unlikely scenario *in vivo* considering the expense of AdoCbl biosynthesis.^17^ We can also conclude that the head-to-tail dimers are able to pre-assemble in a form that can subsequently bind AdoCbl as an additional route to the formation of the active complex.

### AdoCbl binding to TtCarH triggers oligomerization

The mass spectral data presented above have provided good evidence for the arrangement of protein units and chromophores that are necessary for AdoCbl binding and domain assembly. These are equilibrium measurements, however, and questions remain about the pre-equilibrium mechanism. To examine this, we conducted time-resolved fluorescence measurements using stopped-flow spectroscopy. When AdoCbl is titrated into a sample of TtCarH, the protein emission following excitation at 280 nm (predominantly from the five tryptophan residues in each TtCarH monomer, Fig. S12a†) is significantly quenched (Fig. S12b, c and S13a and Table S1†). Because of the spectra overlap (Fig. S12b†) between the tryptophan emission and AdoCbl absorption when bound to TtCarH, we predicted that Förster resonance energy transfer (FRET) between the two chromophores will be the dominant quenching mechanism and that the emission from tryptophans within ∼40 Å of each AdoCbl is likely to be quenched with reasonable efficiency (Fig. S12c and Table S1†). As will become apparent, this quenching provides a useful means of probing the kinetics and mechanism of chromophore binding and oligomerization following rapid mixing of B_12_ species and apo-TtCarH in a stopped-flow (Fig. S13b†).

**Fig. 4:**
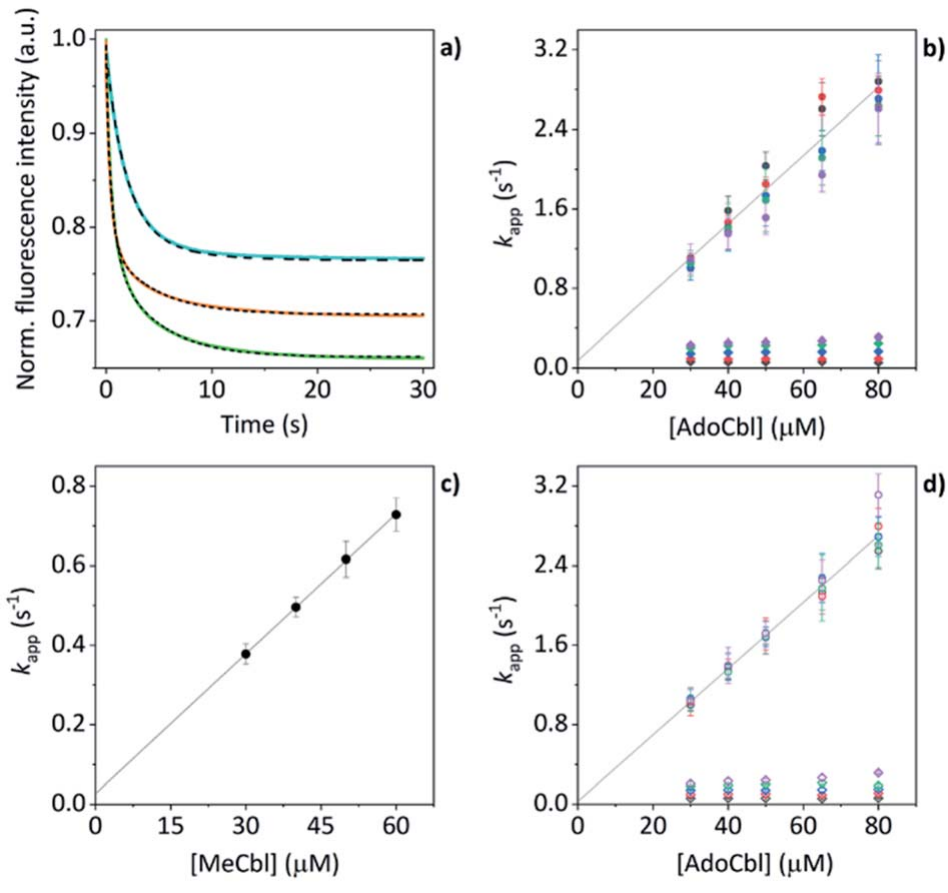
(a) Example stopped-flow traces (solid lines) and corresponding fits (dashed lines) of the fluorescence quenching that follows the rapid mixing of: WT TtCarH *vs.* AdoCbl (green); WT *vs.* MeCbl (blue); G192Q *vs.* AdoCbl (orange). In each case: [protein] = 5 μM; [B_12_] = 60 μM. (b) Apparent rate (*k*_app_) as a function of [AdoCbl] for a range of [WT TtCarH] (see Table S3†). *k*_1_ is [AdoCbl]-dependent and the data from all [TtCarH] were concatenated and fit linearly. *k*_2_ is not [AdoCbl]-dependent. (c) *k*_app_ as a function of [MeCbl] for WT TtCarH and fit linearly. (d) As panel (b) in all aspects but data are from the G192Q variant.

When WT TtCarH is rapidly mixed with a ≥ten-fold excess of AdoCbl (*i.e.*, pseudo-first order conditions) the fluorescence signal is quenched over the course of ∼30 s (Fig. 4a, green trace). The data reveal two kinetic phases, the first of which has an apparent rate (*k*_app_) that is linearly dependent on the concentration of AdoCbl (Fig. 4b). It thus represents a bimolecular reaction involving AdoCbl – *i.e.*, its binding to apo-TtCarH – which occurs with a second-order rate coefficient of 34.3 ± 1.4 s^−1^ mM^−1^. By contrast, the *k*_app_ of the second, slower phase is independent of AdoCbl concentration (Fig. 4b) and presumably therefore corresponds to protein domain assembly steps. If so, this clearly shows that AdoCbl binding drives the oligomerization of TtCarH.

This proposal is supported by the equivalent data from mixing of apo-TtCarH with excess MeCbl. Here, the fluorescence quenching only follows a single phase (Fig. 4a, blue trace), the *k*_app_ of which is dependent on MeCbl concentration (Fig. 4c). The amplitude of this single phase is also smaller than that following AdoCbl binding. These observations are consistent with the data only corresponding to the binding of MeCbl to apo-TtCarH with little subsequent protein domain assembly (and corresponding quenching), as is expected. In fact, the quenching amplitude for MeCbl is smaller than that of the first phase from the AdoCbl data, which corresponds to chromophore binding. This is almost certainly because the close steric contact between Ado and W131 (Fig. S1b†) and any stabilizing interactions are absent when MeCbl is bound. One might expect the lesser steric bulk of the upper axial ligand of MeCbl would make binding more rapid, but the second order rate (11.7 ± 0.1 s^−1^) is three-fold slower than for AdoCbl binding. This result instead suggests that the polar Ado aids with binding to the protein, perhaps helping anchor it in the correct position for favorable binding of the corrin macrocycle. The stopped-flow data for the WT protein and AdoCbl resolve only a single kinetic phase for what we assume corresponds to protein assembly steps. One might expect, however, two or more phases for tetramer assembly if it occurs in a stepwise manner. This could indicate one of several things: (i) that AdoCbl binds preferentially to apo-TtCarH_2_; (ii) that, following binding of AdoCbl to apo-TtCarH_1_, the tetramer assembles in a concerted manner; (iii) that the dimer to tetramer step is spectrally silent or cannot be kinetically resolved. These possibilities will be explored below.

### Protein domain assembly is rate-limited by dimerization

We next conducted equivalent stopped-flow measurements with the G192Q variant, which, as we have seen, predominantly forms dimers following AdoCbl binding. Upon mixing with AdoCbl, the fluorescence quenching again follows two kinetic phases with many features in common with the data from the WT protein (Fig. 4a, orange trace). The first phase is again dependent on AdoCbl concentration (Fig. 4d), with a second order rate (33.4 ± 0.8 s^−1^ mM^−1^) that is the same within error as that of the WT protein. The initial binding of AdoCbl is therefore kinetically equivalent for both WT and G192Q variants (as is binding to MeCbl, Fig. S14†). The second phase is also independent of AdoCbl concentration (Fig. 4d), with very similar *k*_app_ to those measured for the WT and is therefore likely to represent protein domain assembly as before.

Interestingly, however, the amplitude of the fluorescence quenching for G192Q is reduced compared to when AdoCbl binds to WT TtCarH but is greater than when MeCbl binds to the WT (Fig. 4a). Looking at the amplitudes of each phase more closely (Fig. S15†), one can see that the amplitudes for the AdoCbl-binding step are very similar between WT and G192Q. The same is not true for the second phase; here, the amplitude for G192Q – where for the vast majority of the population oligomerization stops at the dimer – is roughly half the amplitude for the WT (Fig. S15 and S16†). This strongly suggests that the data can, in part, resolve the protein monomer to dimer step on the one hand and the dimer to tetramer step on the other, based on differences in spectral amplitudes. The similarity between the *k*_app_ of the second phase for each variant is consistent with the monomer to dimer step being rate-limiting and the dimer to tetramer step being relatively rapid; so much so that, although spectrally resolved, it is not kinetically resolved.

### The TtCarH tetramer assembles predominantly *via* a 1 → 2 → 4 stepwise mechanism

**Fig. 5:**
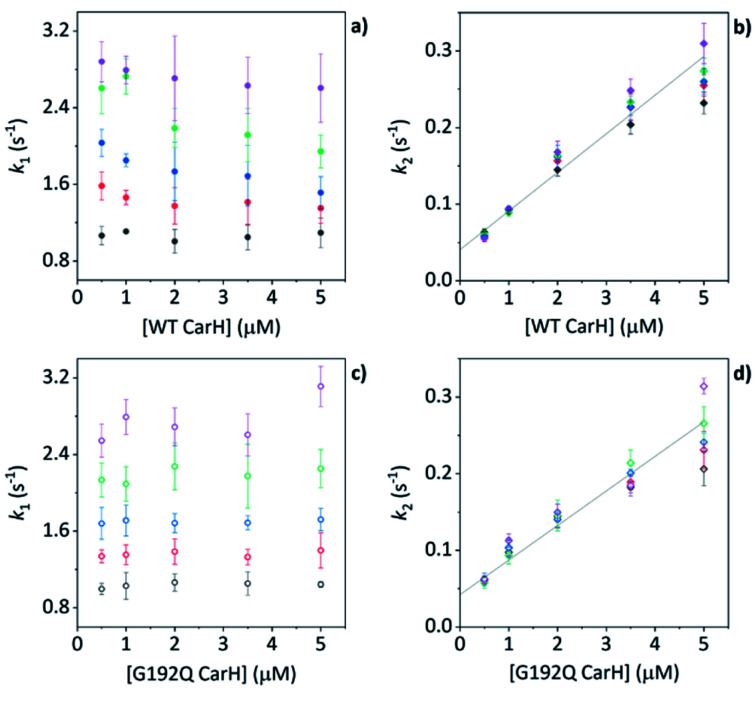
(a, b) *k*_app_ as a function of [WT TtCarH] for a range of [AdoCbl] (see Table S3†). *k*_1_ is not [WT]-dependent (a). *k*_2_ is [WT]-dependent and the data from all [AdoCbl] were concatenated and fit linearly (b). (c and d) As panel (a and b) in all aspects but data are from the G192Q variant.

Before coming to any firm conclusions about the kinetics and mechanism of protein domain assembly, we first need to confirm that the second kinetic phase arises from the TtCarH oligomerization process. One can only infer this indirectly from the stopped-flow data in Fig. 4 because they are presented as a function of AdoCbl concentration. Because domain assembly is a multi-molecular event involving the protein, the kinetics should therefore be dependent on the concentration of TtCarH. Stopped-flow data as a function of protein concentration show this to be the case (Fig. 5). The first kinetic phase for both the WT (Fig. 5a) and G192Q (Fig. 5c) variants is independent of protein concentration. Although AdoCbl binding is a bimolecular process involving the protein, the B_12_ species is at saturating concentrations (≥10×) so the independence of the kinetics on the much more dilute protein concentration is to be expected. The kinetics of the second phase by contrast show a strong dependence on protein concentration for each variant (Fig. 5b and d). In both cases, this dependence is not perfectly linear, which is probably caused by a small but significant inner-filter effect at higher protein concentrations (Fig. S17†). Despite this slight artefact, linear fits give a second order rate for the WT (50.4 ± 2.3 s^−1^ mM^−1^) that is slightly faster than for G192Q (45.1 ± 1.9 s^−1^ mM^−1^). If this marginal difference is significant, it might be because the WT data do not reflect a truly second order process and that the non-linearity here is to some extent also due to ‘contamination’ from a higher order process; *i.e.*, dimer to tetramer transition.

Taken together, these stopped-flow data are consistent with a stepwise mechanism, where AdoCbl predominantly binds to apo-TtCarH_1_, which drives the formation of AdoCbl_2_–TtCarH_2_. Owing to the structural changes involved in each of these steps they are both to some extent rate-limiting. The subsequent association of two sets of AdoCbl_2_–TtCarH_2_ to form AdoCbl_4_–TtCarH_4_ is then relatively rapid because no further structural changes are necessary.

## Discussion

There is mounting evidence that AdoCbl binds to various genetic control elements to achieve transcriptional regulation and that this is widespread in prokaryotes.^21^ In both Gram-positive and Gram-negative bacteria, AdoCbl negatively regulates its own biosynthesis independently of light by directly binding to regions of messenger RNA known as riboswitches.^23^ A light-dependent role for AdoCbl became apparent from studies into the regulatory circuits in Gram-negative *Myxococcus xanthus*, which control the stimulation of carotenoid biosynthesis by blue light.^24^ A combination of AdoCbl and CarH from *M. xanthus* (*Mx*CarH) was found to be necessary for the down-regulation of a light-inducible promoter.^5,6^ This repression is relieved under white light in *M. xanthus* but not by red light alone,^6^ consistent with the absorption spectrum of AdoCbl when bound to TtCarH. Elsewhere, it has been demonstrated that B_12_ is an essential element in the control of bacteriochlorophyll biosynthesis in the photosynthetic purple bacteria, *Rhodobacter capsulatus*.^25^ Here, the aerobic photochemistry of AdoCbl results in its binding to the ‘aerobic repressor’ protein AerR, which together act as an anti-repressor to the tetrapyrrole regulator CrtJ.

It is now clear that the function of both CarH and AerR is mediated through changes to protein–protein interactions, which are mediated by both the binding of B_12_ and its photochemistry. The anti-repressor activity of AerR appears to result from the light-triggered formation of an AerR/B_12_/CrtJ complex, which precludes the binding of CtrtJ to the *bchC* promoter.^25^ In contrast, yeast two-hybrid analysis indicates that MxCarH exists as an oligomer in the dark *in vivo*,^5^ and *E. coli* two-hybrid data are consistent with the C-terminal domain of TtCarH (TtCarHCt) self-interacting only in the presence of AdoCbl. SEC reveals that, while TtCarHCt elutes as the holo-tetramer, the H177A variant, which cannot bind any B_12_, elutes as an apo-monomer, even in the presence of excess AdoCbl.^6^ These *in vitro* data confirmed that oligomerization is facilitated *via* direct interaction between AdoCbl and CarHCt. Both *in vivo* and *in vitro*, the formation of CarH tetramers is impaired by exposure to light of wavelengths <600 nm,^6^ consistent with a role for AdoCbl photochemistry.^11^

Here, we present the first detailed, biophysical investigation into the molecular mechanism of how the binding of AdoCbl drives TtCarH oligomerization. Our MD simulations give the first direct evidence that apo-TtCarH does not adopt the correct configuration to form head-to-tail dimers, which was only previously inferred indirectly from the structural data of AdoCbl_4_–TtCarH_4_.^10^ A key mutation of a salt-bridge residue (D201R) at the head-to-tail dimer interface is known to completely abolish tetramer formation in the presence of AdoCbl (*i.e.*, it elutes in SEC as a holo-monomer).^10^ Our simulations reveal that this and other possible stabilizing interactions are also unlikely to form in apo-TtCarH_1_.

**Fig. 6:**
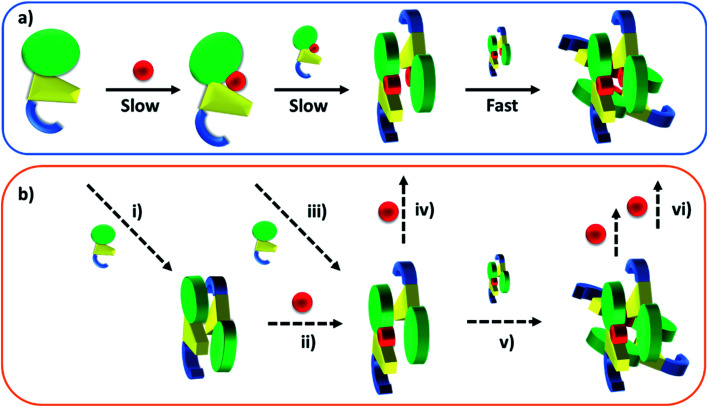
Cartoons illustrating the B_12_-binding and domain assembly events for TtCarH. The domains are color-coded in the same way as Fig. 1a. Red circles are AdoCbl. (a) Proposed, stepwise mechanism under ‘ideal’ conditions, *i.e.*, when AdoCbl is in excess. ‘Slow’ and ‘Fast’ indicate whether a step is rate-limiting or kinetically unresolved, respectively. (b) Additional binding and assembly steps (dashed arrows) that could provide alternative, more convoluted pathways from (a) under conditions where AdoCbl is scarcer: apo-TtCarH_1_ can dimerize to give apo-TtCarH_2_ (i); AdoCbl_1_–TtCarH_2_ can form either by apo-TtCarH_2_ binding one AdoCbl (ii) or by AdoCbl_1_–TtCarH_1_ binding apo-TtCarH (iii); AdoCbl_1_–TtCarH_2_ can then bind another AdoCbl to give AdoCbl_2_–TtCarH_2_ (iv) or dimerize to from AdoCbl_2_–TtCarH_4_ (v); AdoCbl_2_–TtCarH_4_ can bind one or two more AdoCbl to give AdoCbl_3_–TtCarH_4_ and AdoCbl_4_–TtCarH_4_, respectively (vi). There is evidence for each of these intermediates in the mass spectrometry data in Fig. 2 and 3.

Published SEC and analytical ultracentrifugation data are consistent with apo-TtCarH being only a monomer, with no evidence of higher molecular weight species.^8^ Native mass spectrometry has a significantly higher resolution, however, and our data suggest a more nuanced picture (Fig. 2 and 3). We reveal a low population of apo-TtCarH_2_ that appears to be receptive to AdoCbl-binding. Interestingly, apo-CarH from *Bacillus megaterium* has also been shown to self-interact independently of AdoCbl, but to a significantly greater extent than for TtCarH.^9^ apo-BmCarH elutes in SEC as a molten globule (*i.e.*, loosely packed) oligomeric species with a molecular weight consistent with a trimer, although these oligomeric states do not bind DNA. AdoCbl-binding to BmCarH induces tetramer formation, which is much more rigidly structured (and binds DNA *in vivo*) and dissociates into dimers instead of monomers upon illumination. To some extent it appears that TtCarH hedges its bets (Fig. 6): a majority population binds to AdoCbl as apo-TtCarH_1_, but we propose that a minority partially pre-assembles as apo-TtCarH_2_ providing an alternative channel to oligomerization. This inclination to self-associate either in the absence of AdoCbl or at low AdoCbl concentrations is further illustrated by the fact that TtCarH oligomers form with only sub-stoichiometric levels of AdoCbl bound (Fig. 2, 3, S6 and S10†). Although we do not know with precision what AdoCbl concentrations are found in bacteria, it seems unlikely that AdoCbl would be in a significant excess over TtCarH and its concentration might be close to its *K*_D_ for binding to the protein (255 nM).^11^ It is therefore possible that a more fragmentary assembly process is likely under natural conditions, where oligomers form initially with less than a full complement of AdoCbl, before the AdoCbl_4_–TtCarH_4_ state is reached (Fig. 6b; if, indeed, it ever is).

The published structures^10^ of AdoCbl_4_–TtCarH_4_ in the dark reveal a quaternary structure that might suggest a stepwise assembly process. This is borne out by our stopped-flow data (Fig. 4 and 5). We clearly resolved kinetic phases that are consistent first with AdoCbl binding predominantly to apo-TtCarH_1_, which then drives the formation of head-to-tail AdoCbl_2_–TtCarH_2_. To similar extents both steps are rate-limiting to the overall process. It appears that once the corresponding structural changes have occurred, the assembly of thermodynamically stable AdoCbl_4_–TtCarH_4_ is relatively fast; presumably the energy barrier to this final step is low. Our proposed mechanism for chromophore binding and domain assembly for TtCarH is shown in Fig. 6. We present two schemes: Fig. 6a shows a simple mechanism that is likely to take place under ‘ideal’ conditions, where AdoCbl is in a large excess. It shows a 1 → 2 → 4 stepwise assembly mechanism that is consistent with the stopped-flow data and intuitive from the point of view of what is known about the quaternary structure of TtCarH. Fig. 6b shows the more fragmented picture with intermediates supported by the mass spectral data, which might better reflect what occurs under natural conditions when the TtCarH and AdoCbl concentrations are likely to be both similar to *K*_D_.

## Experimental

Experimental details are provided in the ESI along with Fig. S18 and Tables S2 & S3.†

## Author contributions

Conceptualization: ARJ, DJH, SH, and PEB; investigation and data curation: ISC, RB, DJH, LOJ, LAIR, and BB; formal analysis: ISC, RB, DJH, and LOJ with support from all authors; writing – original draft: ARJ, ISC, and RB; writing – review & editing: all authors.

## Conflicts of interest

There are no conflicts to declare.

## Supplementary Material

SC-012-D1SC00522G-s001
