# Supplementary material for: Interplay between chromophore binding and domain assembly by the B12-dependent photoreceptor protein, CarH
Source: Chem Sci. 2021 May 5;12(24):8333–41. doi: 10.1039/d1sc00522g (PMC8221060; doi:10.1039/d1sc00522g)
Supplement: SC-012-D1SC00522G-s001 [file SC-012-D1SC00522G-s001.pdf]

Electronic Supplementary Material (ESI) for Chemical Science.
This journal is © The Royal Society of Chemistry 2021

Interplay Between Chromophore-Binding and Domain Assembly by the B_{12}-Dependent Photoreceptor Protein, CarH

Inês S. Camacho[a,b,c], Rachelle Black[a], Derren J. Heyes[a], Linus O. Johannissen[a], Lennart A. I. Ramakers[a], Bruno Bellina[a,b], Perdita E. Barran*[a,b], Sam Hay*[1] and Alex R. Jones*[a,b,c]

[a] Manchester Institute of Biotechnology and Department of Chemistry, The University of Manchester, 131 Princess Street, Manchester, M1 7DN; [b] Photon Science Institute, The University of Manchester, Oxford Road, Manchester, M13 9PL; [c]Current address: Biometrology, Department of Chemical and Biological Sciences, National Physical Laboratory, Teddington, Middlesex, TW11 0LW, UK

Electronic Supplementary Information

Supplementary Figures

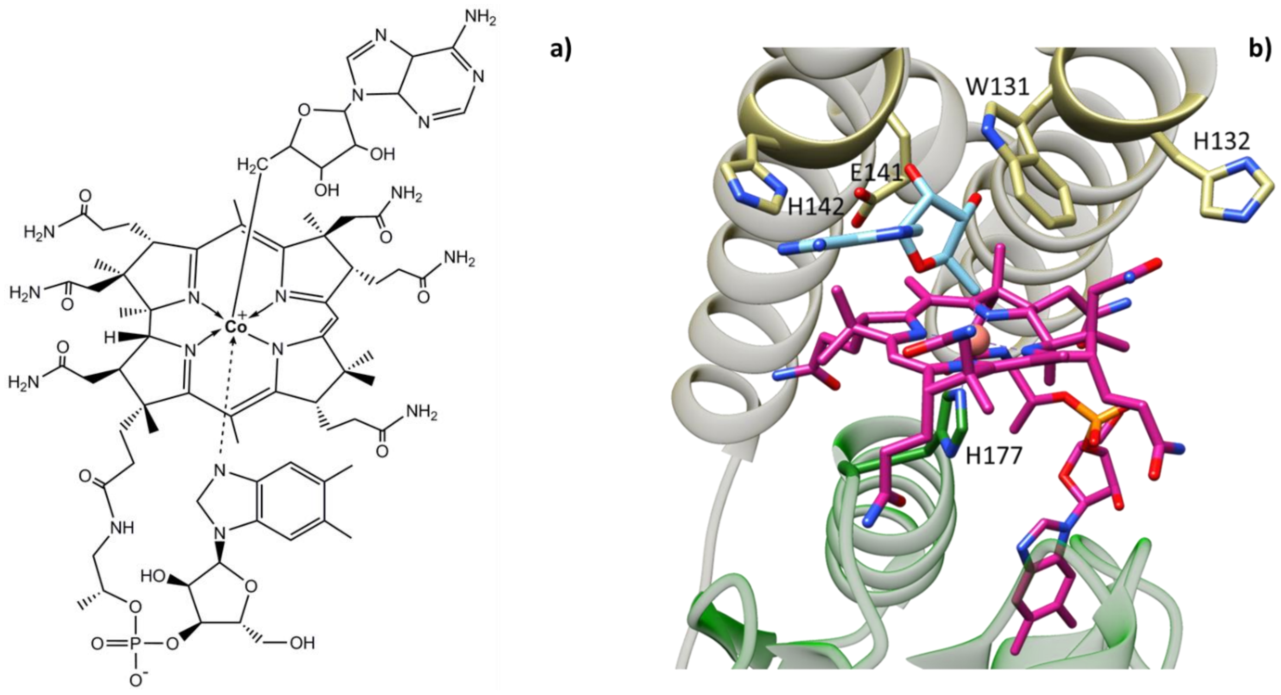

Figure S1a) Chemical structure of AdoCbl in the base-on conformation (with the lower axial coordination site of the central cobalt occupied by the 5,6-dimethylbenzimidazole (DMB) base). **b)** Close-up of the B_{12}-binding site of TtCarH showing AdoCbl in its base-off/His-on conformation. The residues highlighted in yellow are those that contact the upper axial ligand of AdoCbl, Ado (cyan). The cobalt-coordinating His (H177) replacing the DMB base in the lower axial coordination site is highlighted greeb. The domain colors are the same as in Figure 1a.

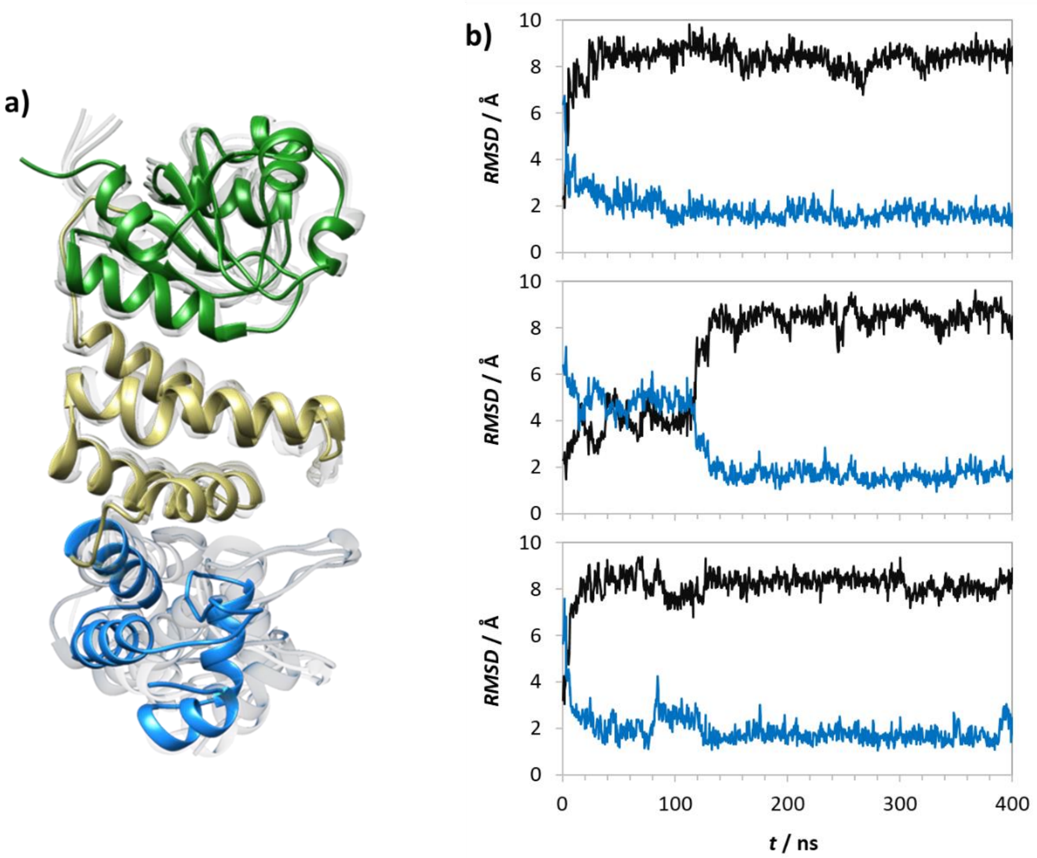

Figure S2a) Representative structures of apo-TtCarH from MD simulations chosen using a single linkage clustering method (see Experimental section). The structures shown cover 95.3% of the total conformational space, with the most populated structure shown in color and the next four most populated clusters represented in grey to illustrate the conformational sampling observed during MD simulations. **b)** Root mean squared deviations (RMSDs) of the simulated apo-TtCarH monomer structure for each of three simulations run in parallel after alignment to the backbone atoms of the starting, photoconverted holo-TtCarH structure (PDB: 5C8F). *Black traces:* RMSD of the protein backbone atoms relative to the starting structure; *blue traces:* RMSD of the protein backbone atoms relative to the average structure across all three simulations. These data suggest that the system reaches equilibration in less than 150 ns in each simulation.

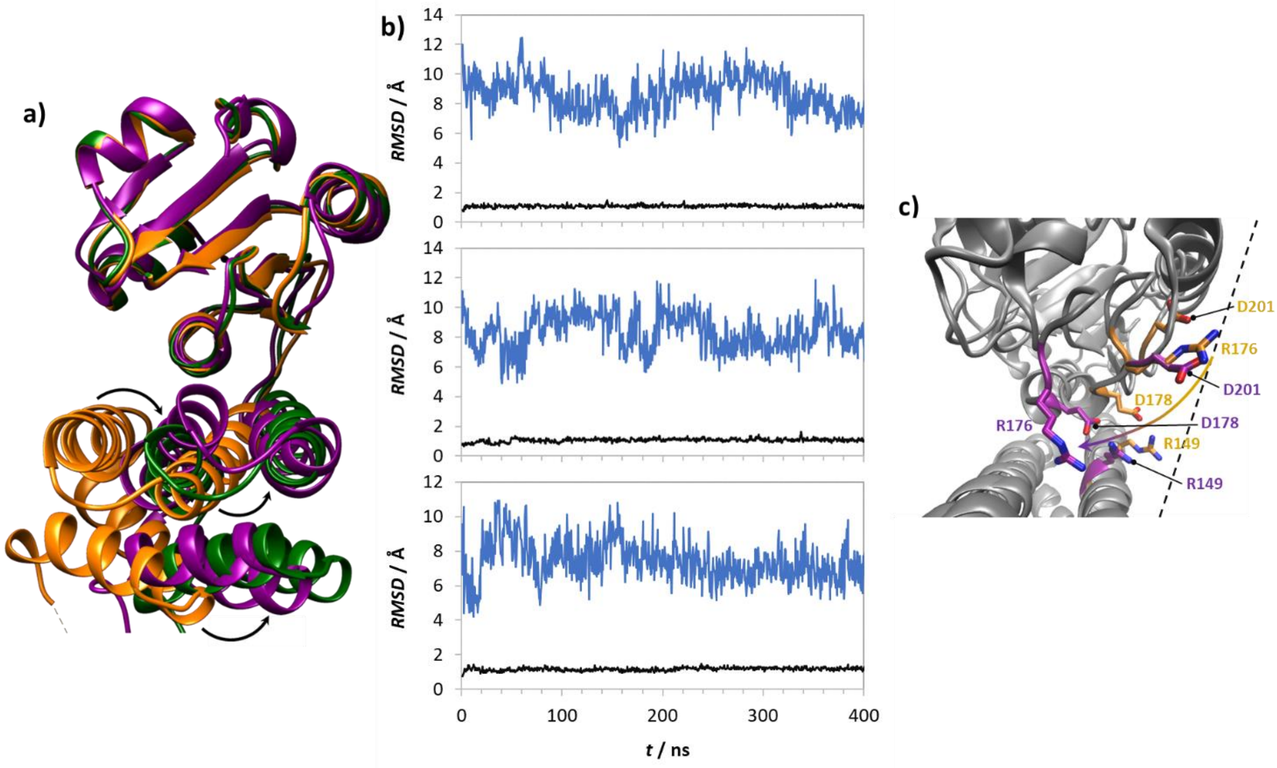

Figure S3a) 4-helix bundle (bottom) positions in each monomer aligned to the B_{12}-binding domain (top): holo-TtCarH (orange, PDB: 5C8D), photo-converted holo-TtCarH (dark green, PDB: 5C8F; *i.e.*, with the upper axial Ado missing) and apo-TtCarH (purple, simulated). Arrows indicate displacement of the 4-helix bundle in photo-converted holo-TtCarH and apo-TtCarH relative to holo-TtCarH. **b)** RMSD of the protein backbone atoms of the apo-TtCarH B_{12}-binding domain (black) and 4-helix bundle (blue) relative to the holo-TtCarH structure after alignment to the B_{12}-binding domain. The average of the blue RMSD traces gives a displacement of the apo-TtCarH 4-helix bundle of 8.14 $\pm$ 1.33 Å relative to the holo-TtCarH structure. For comparison, the RMSD of the 4-helix bundle in the photo-converted holo-TtCarH structure relative to holo-TtCarH after alignment to the B_{12}-binding domain is 9.7 Å, and the RMSD for the photo-converted holo-TtCarH structure relative to the apo-TtCarH is 3.7 $\pm$ 1.2 Å. The 4-helix bundle displacement for both apo-TtCarH and photo-converted holo-TtCarH is therefore of a similar magnitude, but they end up in slightly different positions. **c)** Overlay of monomers from holo-TtCarH and apo-TtCarH but aligned to the 4-helix bundle instead of the B_{12}-binding domain as in Figures 1b-d and S3a. Salt-bridge residues identified here (D178 & R149) and previously[1] (D201 & R176) are highlighted for each monomer in orange and purple, respectively. In combination with Figure 1d, this image serves to illustrate that, whatever domain the structures are aligned to, the residues in the apo-TtCarH monomer are no longer in a position relative to the dimer interface (approximately indicated by the dashed line) to form stabilising salt-bridges. This shifting away from the dimer interface between the holo-TtCarH and apo-TtCarH structures is highlighted by the curved arrow.

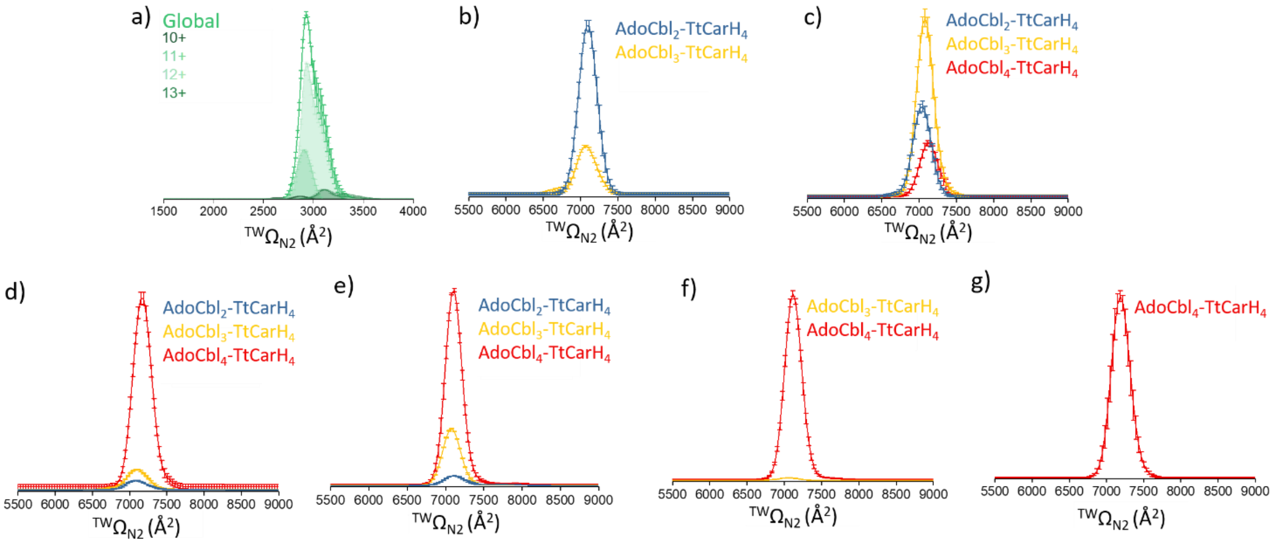

Figure S4. The collision cross section ($^{TW}CCS_{N2}$, $^{TW}\Omega_{N2}$) distributions from ion mobility data of the apo-monomer species **(a)** and of all tetramer species **(b-g)** observed at charge states 23+ to 26+ for WT TtCarH:AdoCbl ratios: 1:0.15, 1:0.25, 1:0.5, 1:0.75, 1:1 and 1:2, respectively. All were performed in triplicate. **a)** The apo-monomer distrubution remains unchanged with increasing proportions of AdoCbl. The charge states ranging from 10+ to 12+ all sit within a very similar range whereas $^{TW}CCS_{N2}$ distribution of 13+ appears to indicate a slightly more unfolded state. **b-g)** Three different tetramer species are evident – AdoCbl$_2$-TtCarH$_4$, AdoCbl$_3$-TtCarH$_4$ and AdoCbl$_4$-TtCarH$_4$ – with relative populations that vary across the range of ratios. Each tetramer returns the same $^{TW}CCS_{N2}$ distribution within error, however, regardless of the number of AdoCbl bound. This suggests that all tetramer structures are highly similar.

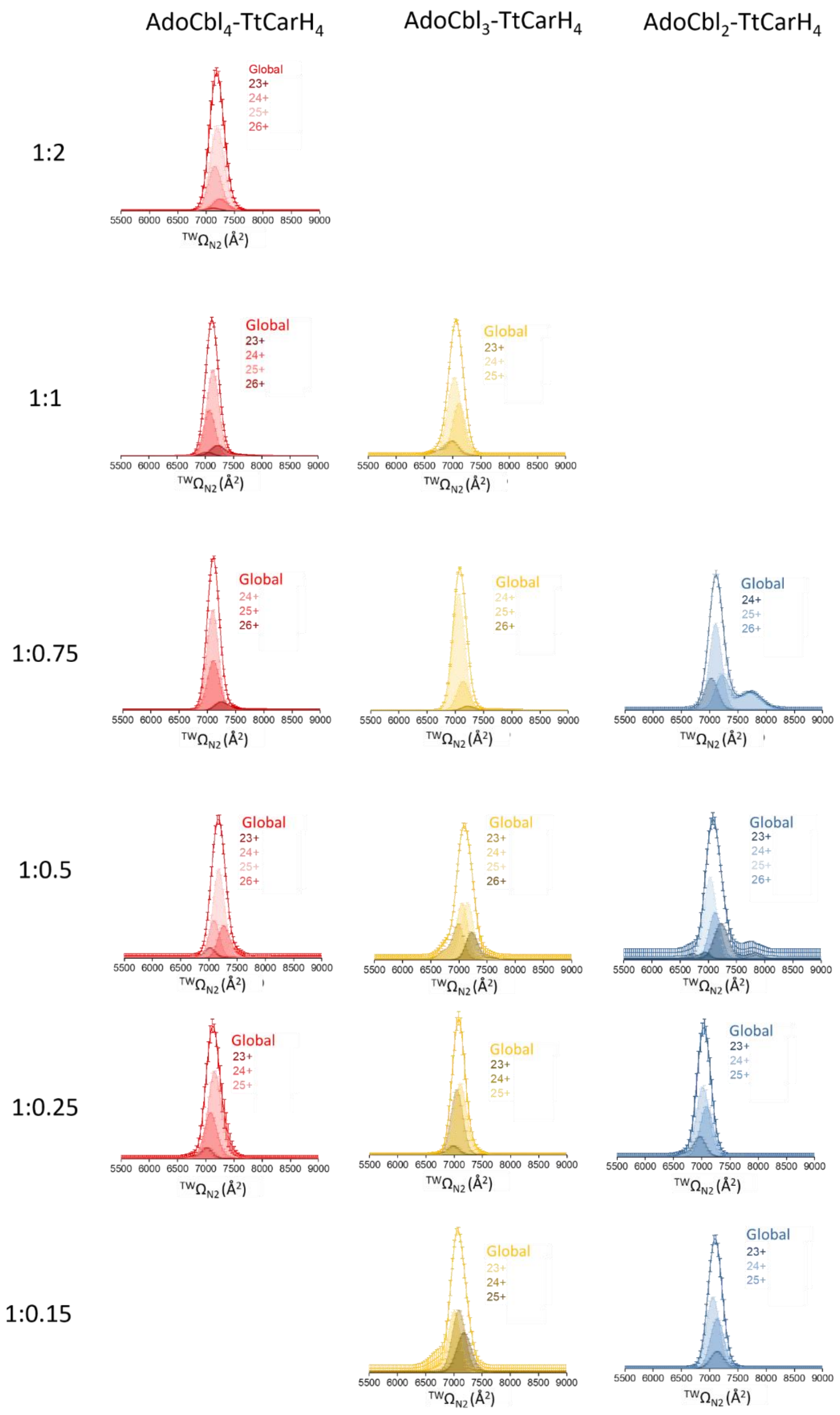

Figure S5. $^{TW}CCS_{N2}$ ($^{TW}\Omega_{N2}$) distributions for all charge states of each tetrameric species ($AdoCbl_4$-$TtCarH_4$ – red; $AdoCbl_3$-$TtCarH_4$ – yellow; $AdoCbl_2$-$TtCarH_4$ – blue) at the different WT TtCarH:AdoCbl ratios (indicated on the left). For each species at each ratio, the global $^{TW}CCS_{N2}$ is shown as a colored line with the different charge states displayed beneath in gradient shades. Data for each charge state have been corrected for their m/z peak height and peak area and were then summed together to give the global $^{TW}CCS_{N2}$. There is little change in the distribution of $AdoCbl_4$-$TtCarH_4$ with increasing AdoCbl content implying that the global 3D structure remains similar throughout. There is slightly greater fluctuation between charge states for $AdoCbl_3$-$TtCarH_4$ and $AdoCbl_2$-$TtCarH_4$. This could be because these forms adopt slightly less rigid structures than $AdoCbl_4$-$TtCarH_4$, but the reduction in the species signal intensity these sub-populations undergo with increasing AdoCbl might also contribute.

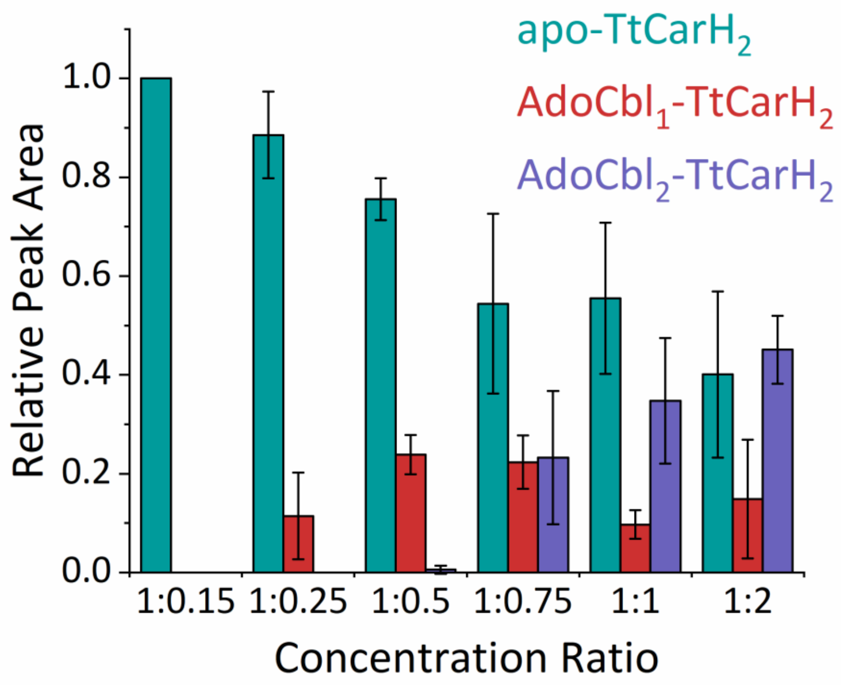

Figure S6. The relative peak area as a function of WT TtCarH:AdoCbl ratio for the low amplitude mass spectral signals between 3500 - 4500 m/z in Figure 2a that correspond to WT TtCarH dimer species. For the sake of simplicity, these peaks are not highlighted on the mass spectrum in Figure 2a with colored, dashed lines as for the tetramer species. Three dimer species are observed – apo-$TtCarH_2$, $AdoCbl_1$-$TtCarH_2$ and $AdoCbl_2$-$TtCarH_2$ – the populations of which vary across the range of ratios. At a ratio of 1:0.15, there is only apo-$TtCarH_2$ present. When compared to the equivalent plots for the tetramers (Figure 2a), these data suggest that any $AdoCbl_1$-$TtCarH_2$ and $AdoCbl_2$-$TtCarH_2$ that form at this ratio rapidly combine to form tetrameric species. This trend appears to continue. Although increasing populations of $AdoCbl_1$-$TtCarH_2$ and $AdoCbl_2$-$TtCarH_2$ are evident at higher AdoCbl concentrations, they always have a lower relative population than the equivalent tetramers ($AdoCbl_2$-$TtCarH_4$, $AdoCbl_3$-$TtCarH_4$ and $AdoCbl_4$-$TtCarH_4$) at the same ratios.

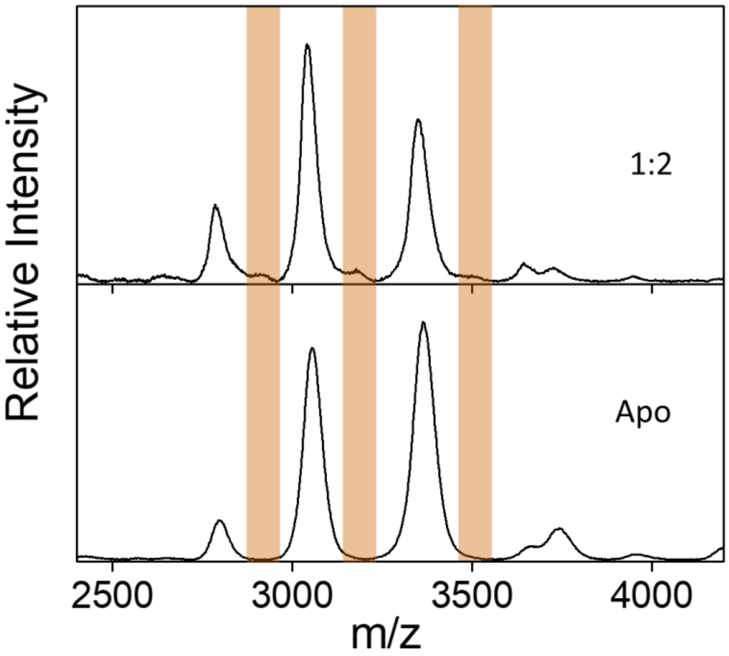

Figure S7. An expansion of the region of the mass spectrum that corresponds to the TtCarH monomer species. Data are compared for a sample of apo-TtCarH (bottom) to data when AdoCbl is in excess (top, *i.e.*, a WT TtCarH:AdoCbl ratio of 1:2) where additional, low amplitude signals (highlighted) are evident. The mass difference is equivalent to AdoCbl (~1550 Da) and these signals therefore correspond to $AdoCbl_1$-$TtCarH_1$. There is no evidence of this signal at WT TtCarH:AdoCbl ratios less than 1:2.

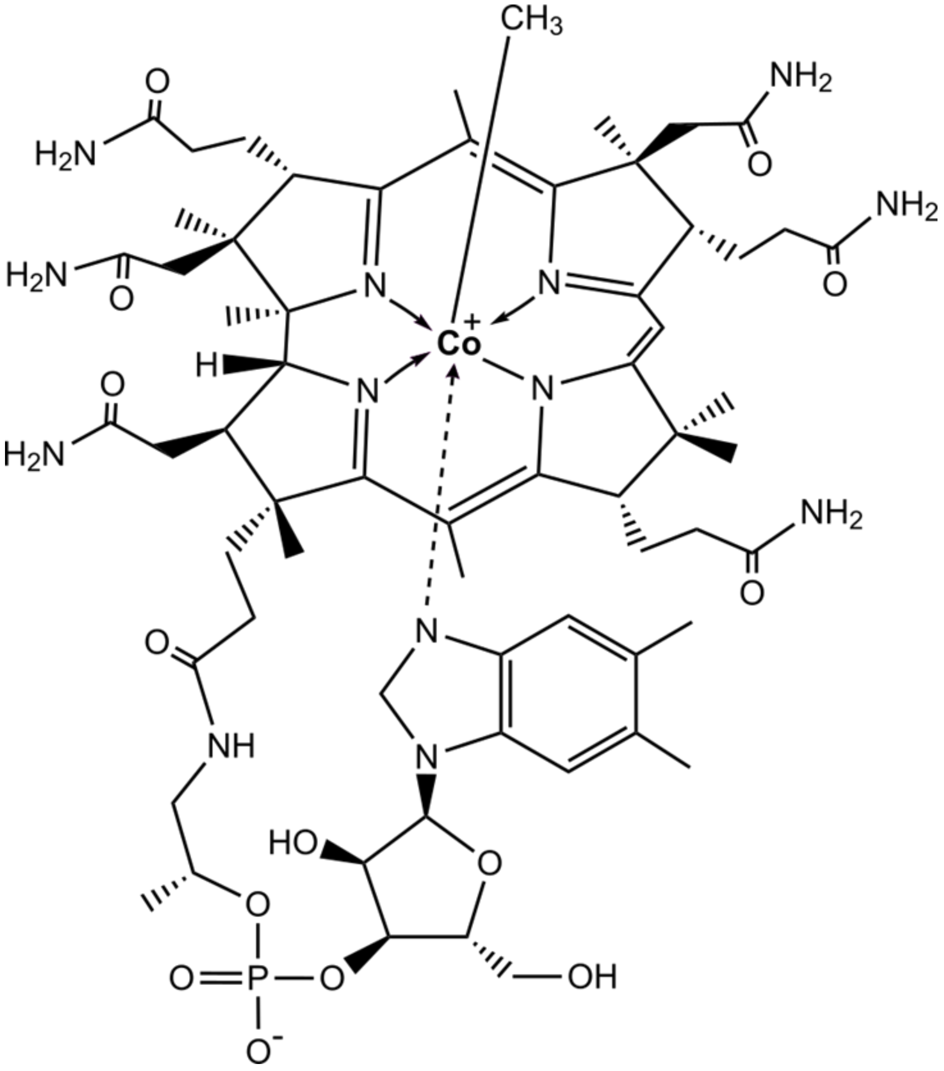

Figure S8. Chemical structure of MeCbl in the base-on conformation.

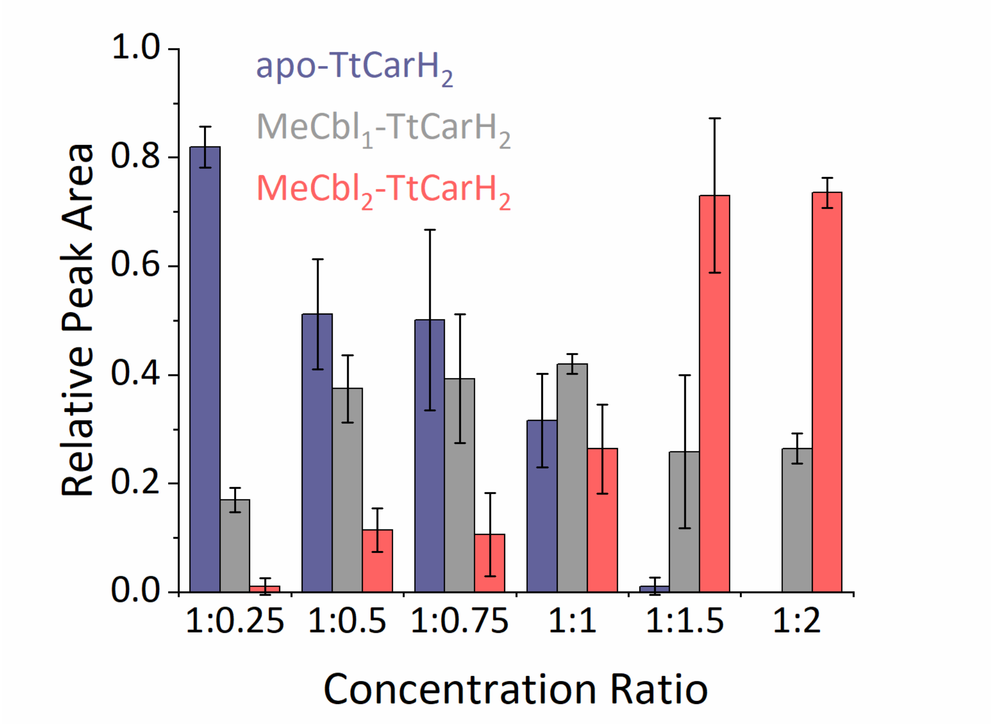

Figure S9. The relative peak area as a function of WT TtCarH:MeCbl ratio for the low amplitude mass spectral signals between 4000 - 5000 m/z in Figure 3a that correspond to dimer species (color matched in Figure 3a with the dashed lines). Much like for AdoCbl (Figure S6), three dimer species are observed – apo-TtCarH$_2$, MeCbl$_1$-TtCarH$_2$ and MeCbl$_2$-TtCarH$_2$ – and again their populations vary across the range of ratios. The lowest MeCbl concentration is also predominantly apo-TtCarH$_2$, but unlike AdoCbl, small but significant populations of the other dimer species are clearly evident. Indeed, relative to apo-TtCarH$_2$, MeCbl$_1$-TtCarH$_2$ and MeCbl$_2$-TtCarH$_2$ become far more prominent with increasing concentration of MeCbl. This is almost certainly because, unlike those binding AdoCbl, the dimers here do not combine to form tetramers and therefore their populations are not depleted in the same way. Nevertheless, all dimer species remain very much minor sub-populations in a spectrum dominated by monomer species (Figures 3a&b).

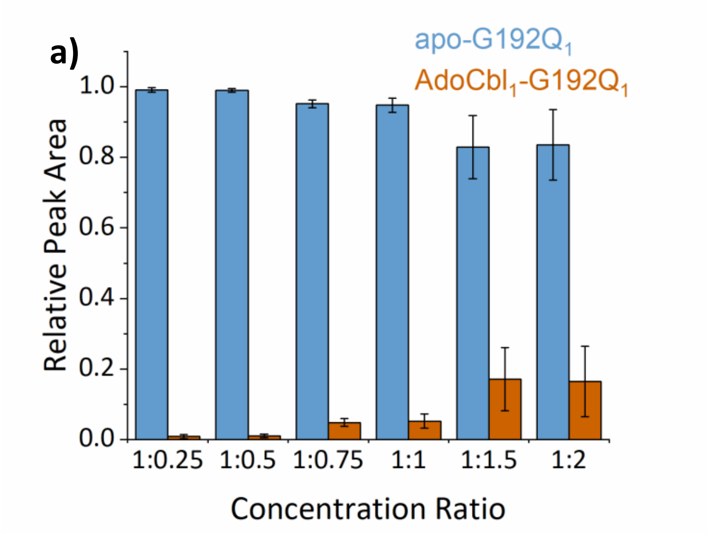

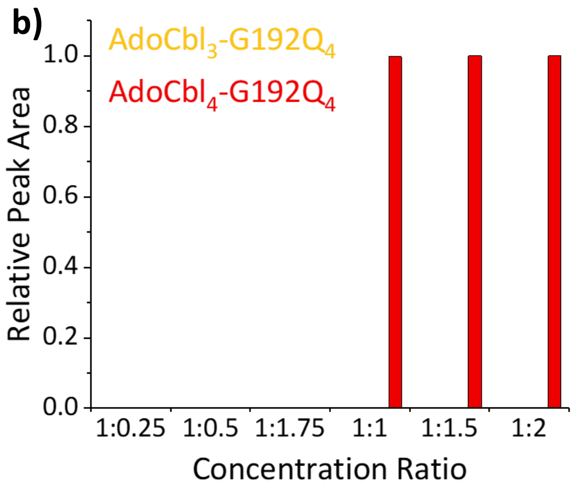

Figure S10. The relative peak area as a function of G192Q:AdoCbl ratio for the mass spectral signals in Figure 3c that corresponds to monomer **(a)** (color matched in Figure 3c with the dashed lines) and tetramer species **(b)** (not highlighted in Figure 3c, for the sake of simplicity, but the low amplitude signals are visible between 5500 – 6500 m/z). **a)** Two monomer species are observed – apo-G192Q_1 and $AdoCbl_1$-G192Q_1 – the populations of which vary across the range of ratios. Like for WT TtCarH (Figure S6), apo-G192Q_1 is by far the most significant monomer species across all ratios. This is likely to reflect the dominant dimer species (Figures 3c&d) being more stable and that most $AdoCbl_1$-G192Q_1 that does form combines to form $AdoCbl_1$-G192Q_2 or $AdoCbl_2$-G192Q_2. Unlike WT TtCarH, however, where there is only the slightest hint of $AdoCbl_1$-$TtCarH_1$ when the AdoCbl is in excess (Figure S7), $AdoCbl_1$-G192Q_1 is evident even at the lowest AdoCbl concentrations. The dominant G192Q dimers are therefore not as stable as the corresponding dominant tetramer for WT TtCarH, which draws the position of equilibrium further away from the holo-monomer. **b)** Although published SEC data suggest G192Q does not form tetramers,[1] a small sub-population is clearly evident in Figure 3c at ratios >1:1. Unlike when AdoCbl is bound to WT TtCarH, there is only one tetramer population, $AdoCbl_4$-G192Q_4, which only appears at AdoCbl concentrations where $AdoCbl_2$-G192Q_2 becomes the predominant species (Figure 3d). The G $\rightarrow$ Q substitution is thought to sterically hinder the native association between pairs of head-to-tail dimer, which means these tetramers could be the result of non-specific association between $AdoCbl_2$-G192Q_2. This is supported by the fact that for the WT, tetramers are observed even at the low [AdoCbl] concentrations. The $^{TW}CCS_{N2}$ for both the $AdoCbl_4$-$TtCarH_4$ and the $AdoCbl_4$-G192Q_4 are compared in Figure S11b.

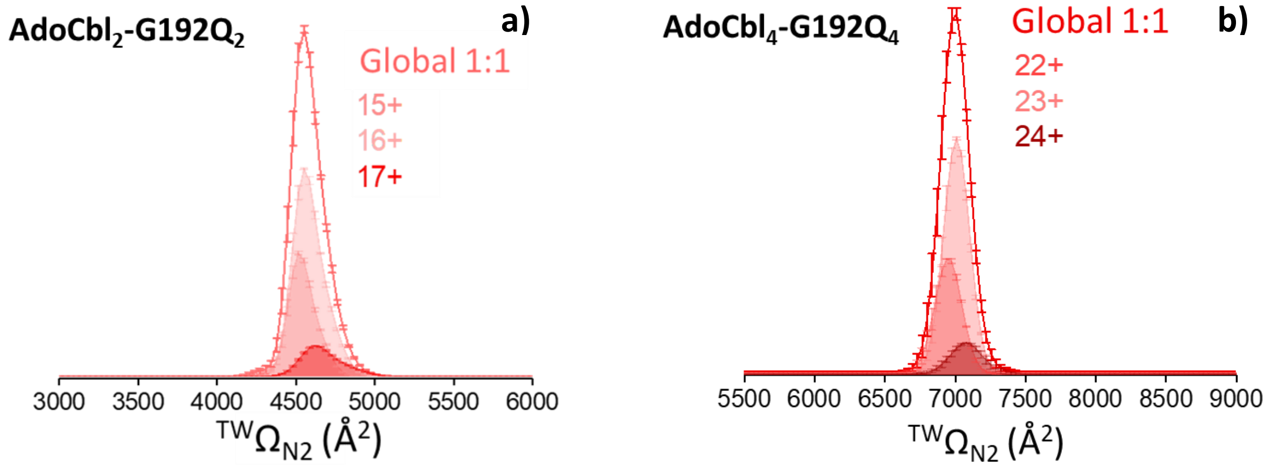

Figure S11. $^{TW}CCS_{N2}$ ($^{TW}\Omega_{N2}$) distributions from ion mobility data of AdoCbl$_2$-G192Q$_2$ **(a)** and AdoCbl$_4$-G192Q$_4$ **(b)** for a G192Q:AdoCbl ratio of 1:1. For each species, the global $^{TW}CCS_{N2}$ is shown as a colored line with the different charge states displayed beneath in gradient shades. Data for each charge state have been corrected for their m/z peak height and peak area and were then summed together to give the global $^{TW}CCS_{N2}$. **a)** The 15+ and 16+ states for AdoCbl$_2$-G192Q$_2$ each give similar $^{TW}CCS_{N2}$. The minor 17+ state is slightly broader and hence has more conformation flexibility. **b)** The AdoCbl$_4$-G192Q$_4$ distribution follows a very similar pattern. AdoCbl$_4$-G192Q$_4$ adopts a slightly smaller global $^{TW}CCS_{N2}$ than AdoCbl$_4$-TtCarH$_4$ (Figure 2c), perhaps indicative of non-specific association, this marginal (1.6%) difference is within instrumental error.

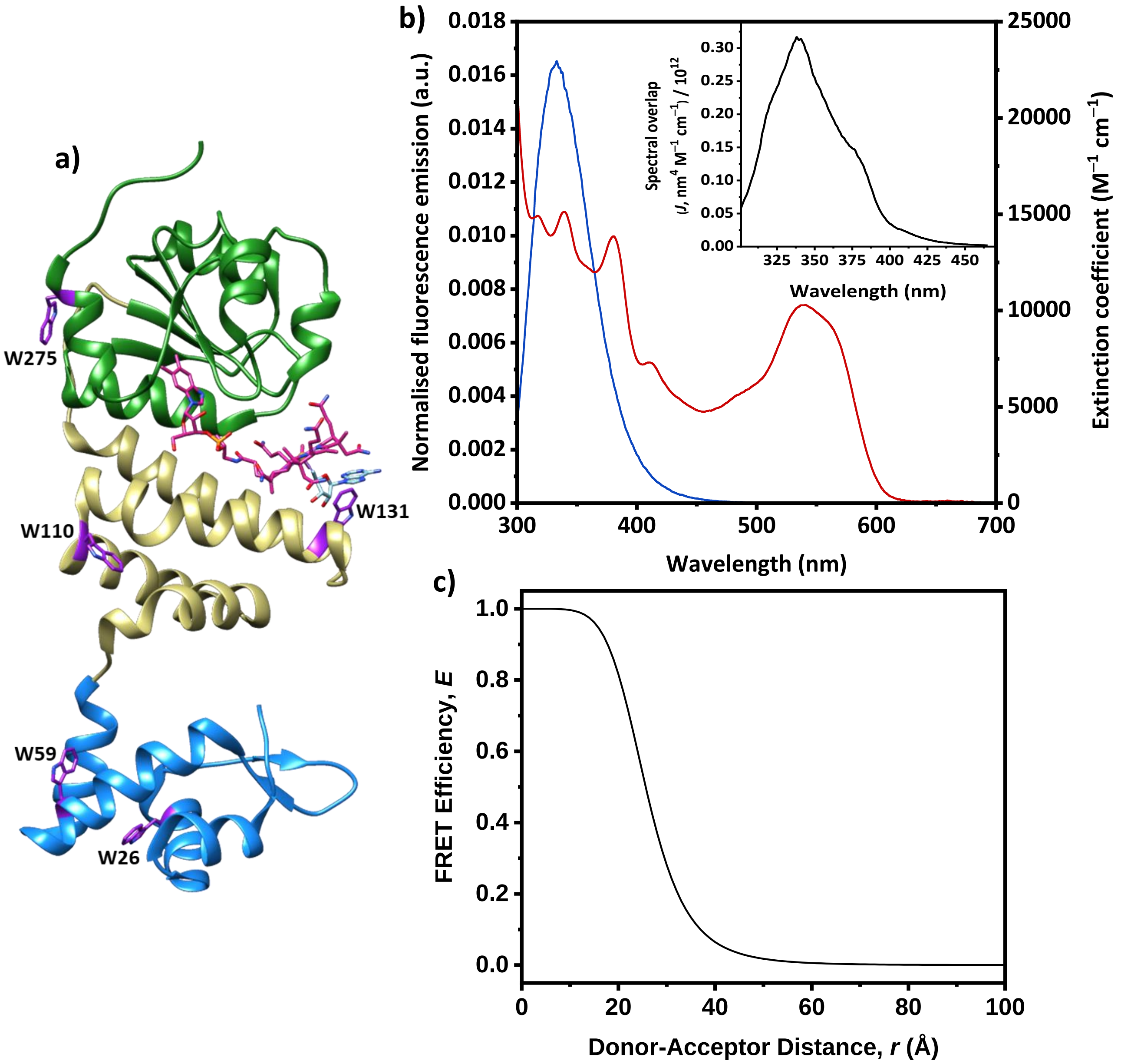

Figure S12a) A monomer unit from holo-TtCarH (PDB: 5C8D) with the five tryptophan residues (purple) and bound AdoCbl (magenta) highlighted. **b)** Absorbance spectrum of the AdoCbl in holo-TtCarH (red) and the normalized fluorescence emission spectrum of apo-TtCarH (blue, excitation at 280 nm); inset: spectral overlap (*J*) calculated from the spectra in the main panel using the Förster equation.[2] A Förster distance, R_0 = 26 Å, was calculated from the spectral overlap assuming κ^2 = 2/3 and the refractive index, *n* = 1.4. A florescence quantum yield, *Q* = 10%, was used for the tryptophan emission. If *Q* = 1% or 30% - reasonable lower and upper bounds for tryptophan fluorescence[3] - the resulting R_0 = 18 and 31 Å, respectively. **c)** FRET efficiency (*E*) as a function of donor (tryptophan) - acceptor (AdoCbl) distance (*r*), calculated[2] using R_0 = 26 Å. One would therefore expect some extent of quenching by AdoCbl of tryptophans within ~ 40 Å (see Table S1).

Table S1. Distances between the Cα in each of the 20 tryptophans (W) in the entire holo-TtCarH tetramer (*i.e.*, 5 in each monomer, Figure S12a) and the Co ion of AdoCbl in monomer A. Distances were measured using the published[1] crystal structure (PDB: 5C8D) and each monomer of the tetramer is given a letter designation A-D, with A/B and C/D being the two head-to-tail dimers. There will, of course, be equivalent and corresponding distances between each W and the AdoCbl in monomers B-D. From Figure S12, we can estimate that fluorescence from W within ~ 40 Å is likely to be quenched with non-negligible efficiency by AdoCbl (highlighted in green below). This includes W from each monomer, which means the fluorescence quenching experiments conducted by stopped-flow spectroscopy (Figure S13) are highly likely to be sensitive to both AdoCbl-binding and the subsequent tetramer domain assembly steps.

Tryptophan and Monomer	Distance (Å)
W131.A	9.63
W59.D	25.58
W110.A	26.22
W131.B	28.10
W275.A	32.53
W110.C	34.15
W275.B	35.69
W110.D	35.94
W26.D	36.70
W110.B	38.51
W275.C	42.09
W59.C	44.40
W26.A	44.83
W59.A	46.57
W26.C	46.78
W275.D	49.73
W131.D	55.28
W26.B	57.05
W131.C	57.31
W59.B	59.42

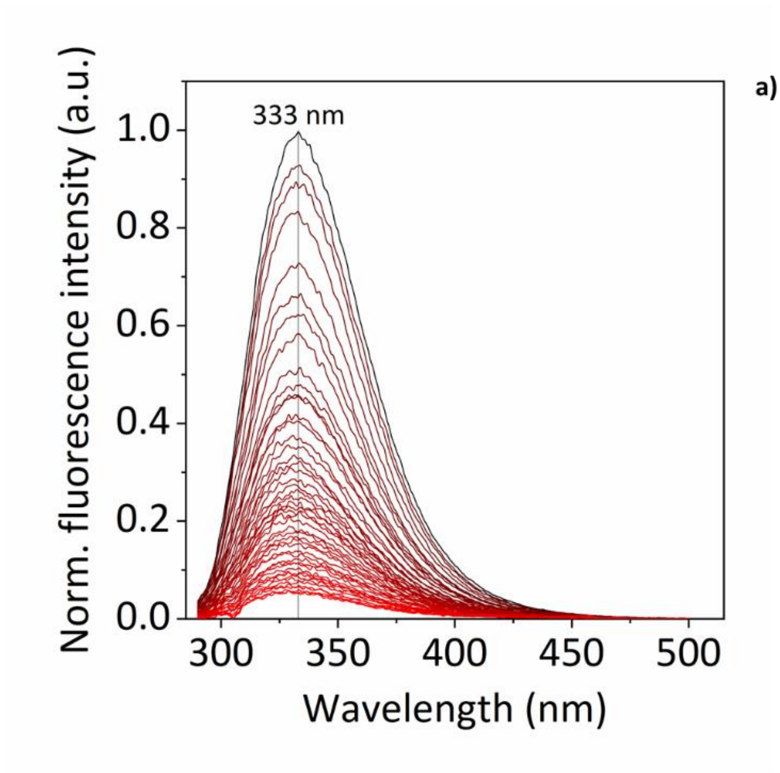

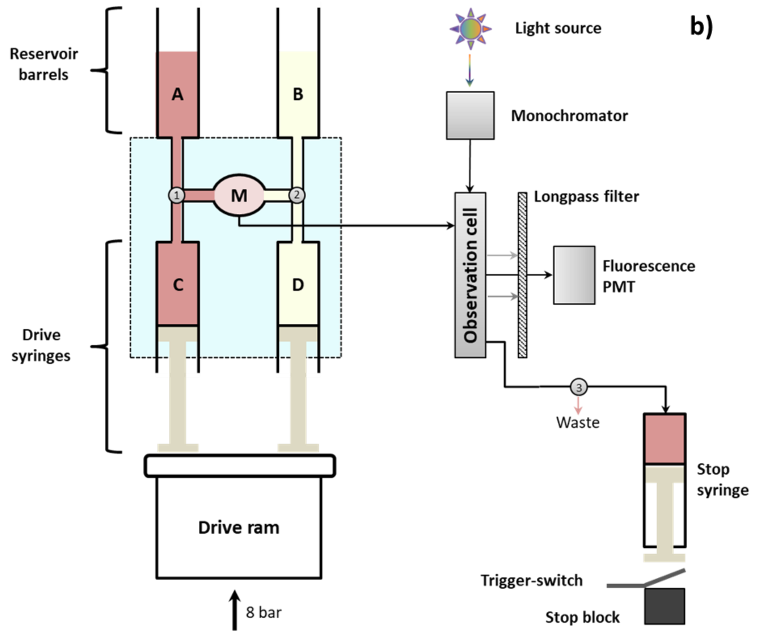

Figure S13a) Spectral evolution of the fluorescence (λ_{exc} = 280 nm) from TtCarH during titration with increasing [AdoCbl]. This fluorescence is significantly quenched following AdoCbl binding and thus changes to the fluorescence signal that result from rapid mixing of TtCarH and AdoCbl enable us to monitor the B_{12}-binding and TtCarH domain assembly mechanisms. Small increments (10 µL) of AdoCbl (up to 30 µM) were sequentially added to a sample of 6 µM WT apo-TtCarH, with fluorescence emission spectra acquired following each addition. Each spectrum is corrected for dilution. **b)** Schematic representation of the SX20 stopped-flow spectrometer (*Applied Photophysics*) used in all transient measurements. A and B, reservoir barrels; C and D, drive syringes; M, mixing chamber; PMT, photomultiplier tube. The system valves are represented by greyed numbered circles. TtCarH (syringes A/C) and AdoCbl (syringes B/D) were rapidly mixed together in the 20 µL observation cell.

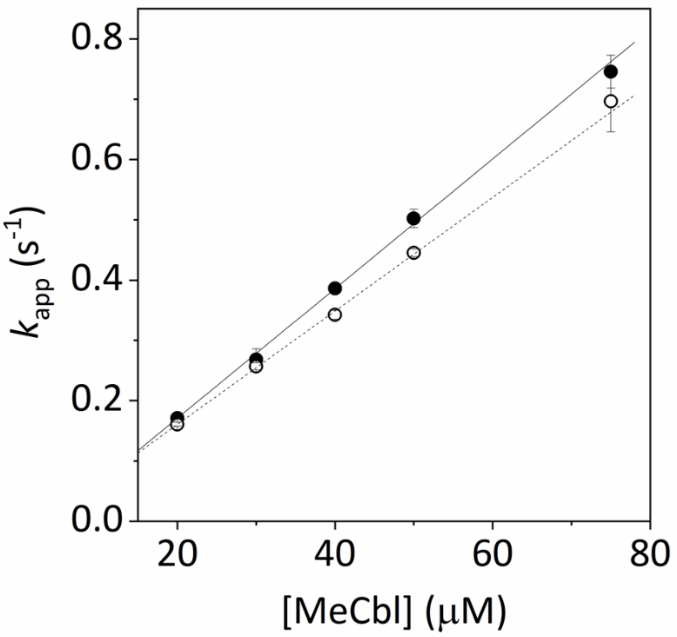

Figure S14. Comparison of the apparent rate constant (k_{app}) of WT (solid circles) and G192Q (open circles) TtCarH variants as a function of MeCbl concentration, at a fixed protein concentration of 5 μM. In both cases, the fluorescence quenching that follows rapid mixing of protein and MeCbl contains a single kinetic phase that is linearly-dependent on [MeCbl]. The data therefore correspond to the bimolecular binding of MeCbl to protein monomers, with no significant quenching contributions from protein domain assembly steps. The second order rates for WT (10.8 ± 0.1 s^{-1} mM^{-1}) and G192Q (9.4 ± 0.1 s^{-1} mM^{-1}) are very similar.

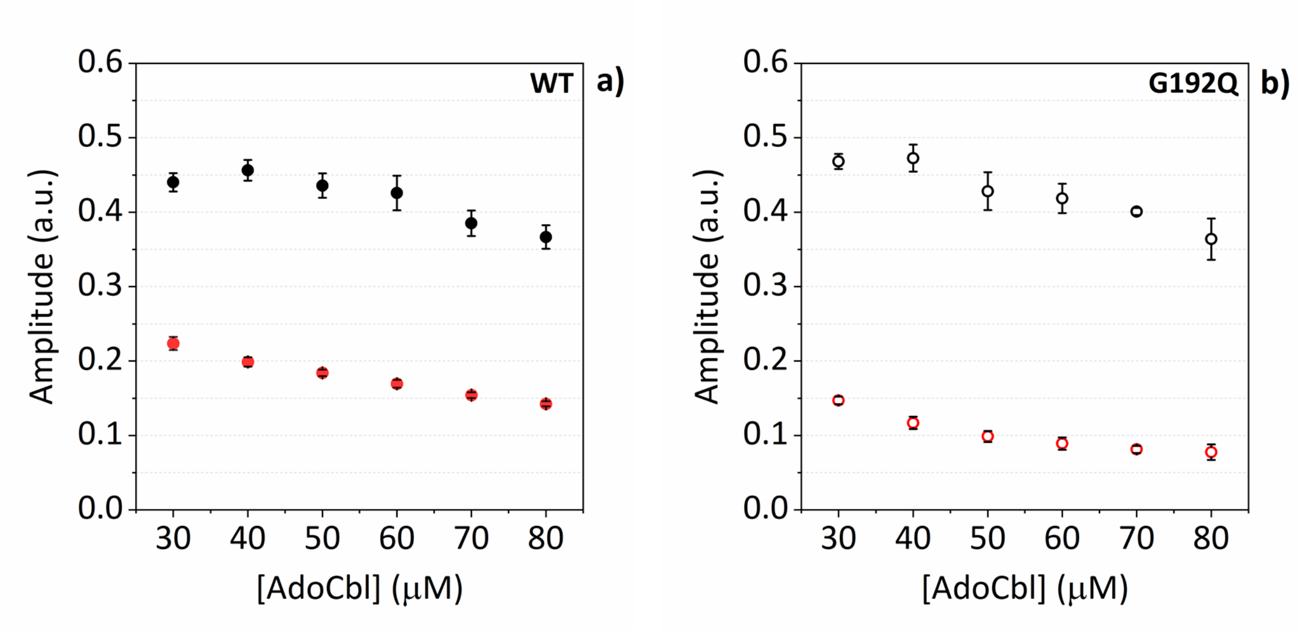

Figure S15. Amplitudes of the different kinetic phases of the fluorescence quenching that follows rapid mixing of AdoCbl with WT TtCarH (filled circles) **(a)** and G192Q (open circles) **(b)** as a function of [AdoCbl]. The amplitudes for the faster kinetic phase (black) are the same within error for each variant, indicating that they represent an equivalent process (*i.e.*, binding of AdoCbl to protein monomers). The amplitudes for the slower kinetic phase (red) are consistently smaller for G192Q than for WT TtCarH. Because this phase is linearly-dependent on [protein] (Figures 4b&d) for both variants, it corresponds to quenching from protein domain assembly. The smaller amplitude for G192Q therefore indicates that there is less quenching than for WT TtCarH, consistent with domain assembly predominantly stopping at the dimer rather than going on to form a significant proportion of tetramers.

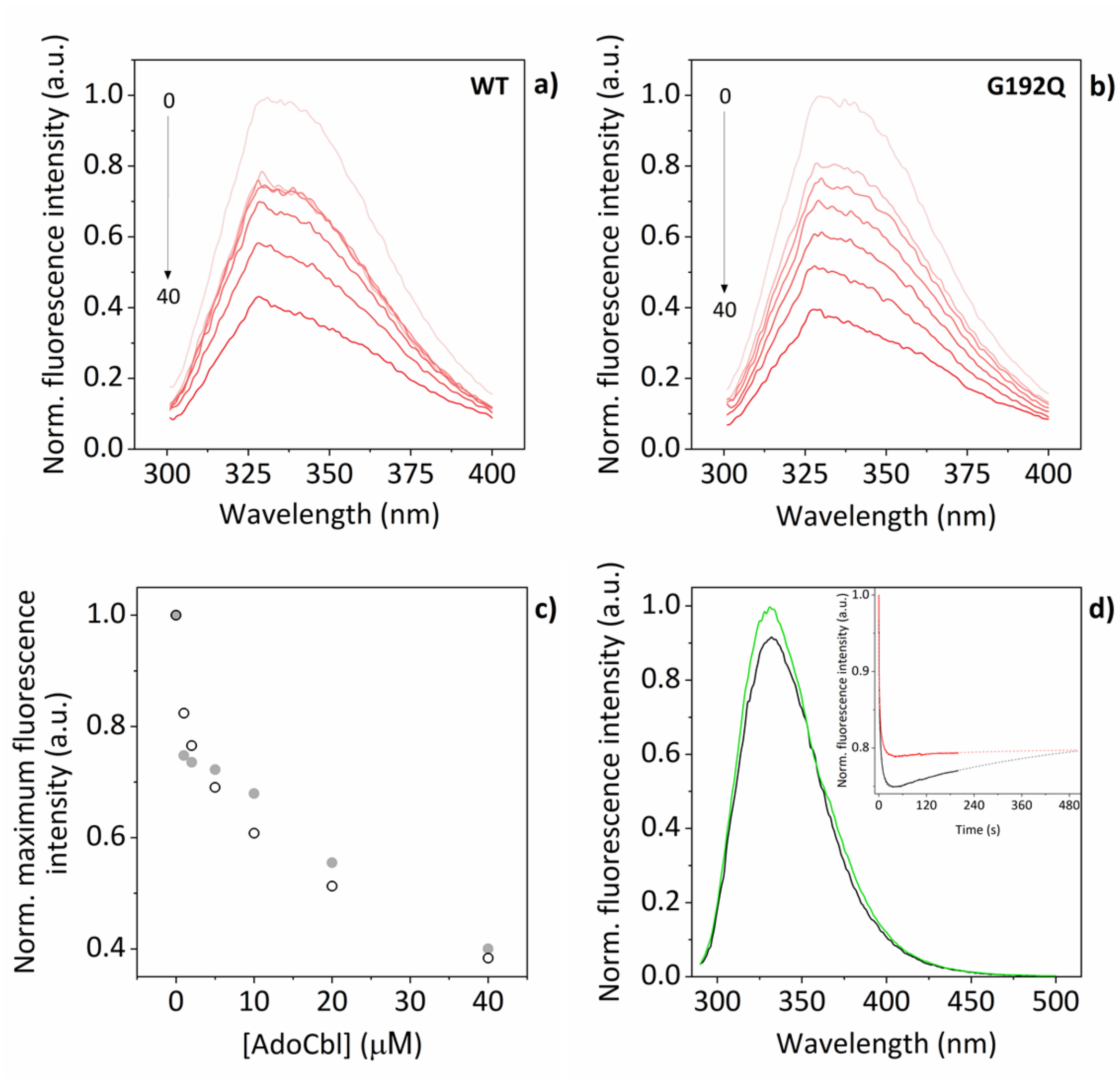

Figure S16. Comparison of fluorescence quenching between TtCarH variants. To further investigate the difference in quenching amplitude observed in the stopped flow data (Figures 4a & S15), static fluorescence spectra (λ_{exc} = 280 nm) from WT TtCarH **(a)** and G192Q **(b)** were acquired during titration with increasing concentrations of AdoCbl (up to 40 μM). **c)** Maximum fluorescence intensity (at 335 nm) as a function of AdoCbl concentration for WT TtCarH (solid circles) and G192Q (open circles). In contrast to the stopped flow data, panels **(a-c)** show no difference in the fluorescence quenching between the two variants. **d)** Emission spectra of WT TtCarH before (black) and after (green) exposure to a 530 nm LED for 5 s. The photo-converted monomer has a larger fluorescence magnitude than the 'dark' state tetramer. Inset: long time-base fluorescence stopped flow traces following rapid mixing of WT TtCarH (solid black line) and G192Q (solid red line) with AdoCbl. In each case, the data were fit to the sum of three exponentials (two negative phases and one positive phase) and extrapolated to longer times (dashed lines) to illustrate convergence. Although the quenching amplitudes in panels **(a-c)** are ostensibly the same, the data in **(d)** reveal this to be an artefact of the measurement. In each case the static spectra where acquired from the same protein sample with sequential additions of AdoCbl, with each TtCarH sample therefore exposed to the excitation light for several minutes. This light was also absorbed by bound AdoCbl over the same period, which thus slowly and irreversibly activated the TtCarH photoreceptor. The static fluorescence spectra for each variant following the titration were the same and dominated by the photoconverted protein.

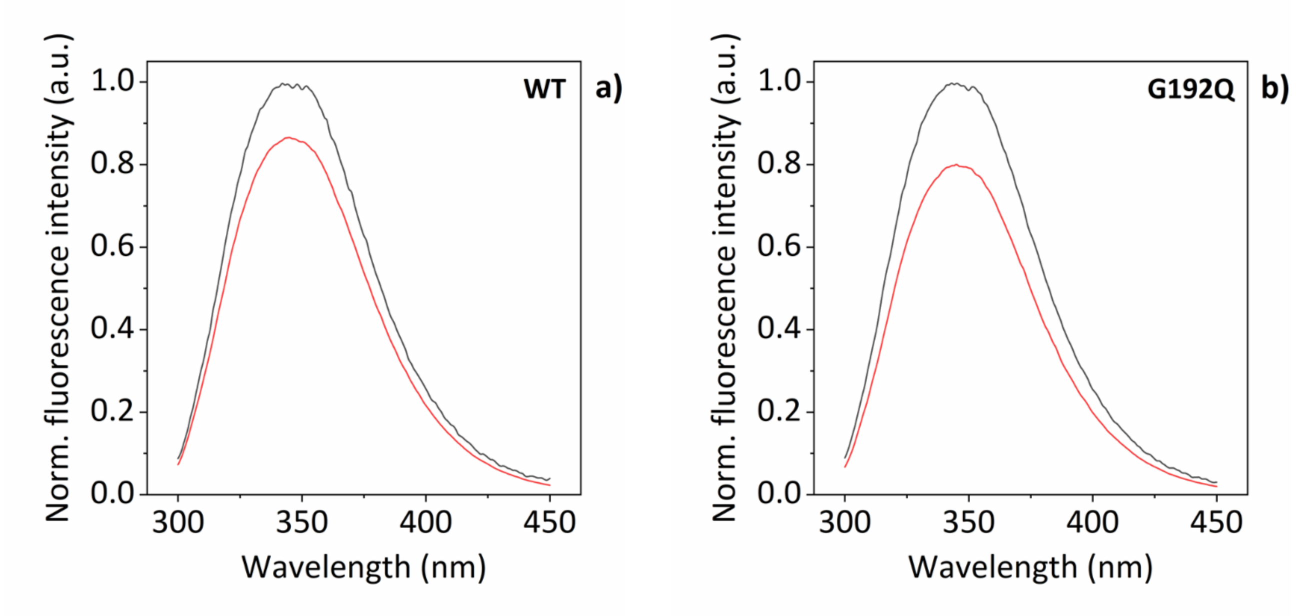

Figure S17. Fluorescence spectra of 0.5 µM (black) and 5 µM (red) apo-TtCarH for the WT **(a)** and G192Q variants **(b)**. To normalise, the 0.5 µM spectra have been multiplied by 10. For both variants, the normalised fluorescence amplitude for the 5 µM protein sample is lower than for the 0.5 µM sample, presumably owing to the inner filter effect.

Experimental Section

Molecular Dynamics Simulations

Molecular dynamics (MD) simulations of apo-TtCarH were performed using the crystal structure[1] of photo-converted TtCarH (PDB: 5C8F) as a starting point, using the Gromacs package[4] with the Amber03 force field,[5] a solvation box of minimum 10 Å around the protein and periodic boundary conditions. Three MD simulations were run in parallel after energy minimization using the following protocol: the system was initially thermalized to 300 K for 100 ps using the *NVT* ensemble (constant volume), and the pressure was then equilibrated for 100 ps using the *NPT* ensemble (constant pressure) with harmonic constraints of force constant 10 kJ mol^{-1} $Å^2$ applied to the protein; constraints and pressure couplings were then switched off and the system relaxed using *NPT* at 250, 280, 290 and 300 K for 1 ns each. Finally, 400 ns of *NPT* dynamics were run at 300 K. Representative structures (Figure S2a) were chosen using a single linkage clustering on the whole protein (not including hydrogen atoms) for all three simulations together, with a cut-off of 0.17 nm (the average RMSD between any two structures being 0.35 nm), which resulted in 95.3% of structures present in the top 5 clusters, which have the following populations: 78.7%, 8.89%, 4.56%, 2.38% and 0.833%.

Materials and Protein Production

Unless otherwise stated, all commercial reagents were obtained from Sigma-Aldrich and used without further purification. Plasmids containing the genes encoding WT and G192Q variants of TtCarH were kindly provided by S. Padmanabhan and Montserrat Elías-Arnanz. The genes had been cloned into modified pET15b (*Novagen*) expression vectors using NdeI and BamHI restriction enzymes, to provide a N-terminal 6xHis affinity tag as previously described.[6]

The following overexpression and purification protocols were used for both WT and G192Q variants of TtCarH. The plasmids were transformed into *E. coli* BL21 (DE3) cells (*Novagen*), and a single colony inoculated into a small volume of selective (Amp^R) LB medium. The cell cultures were grown to an OD_{600} ~ 0.8 at 37 °C and 200 rpm, at which time they were inoculated into a larger volume of fresh LB. The fresh cell cultures were grown to an OD_{600} ~ 0.7 at 37 °C and 200 rpm, at which time they were cooled down to the induction temperature of 25 °C. Protein expression was then induced with 0.5 mM IPTG and the cells were left to incubate overnight at 25 °C and 200 rpm. Cell were then harvested by centrifugation at 5,000 rpm and 4 °C for 20 mins, collected, rapidly frozen in liquid nitrogen, and stored at – 80 °C. For purification, cells were thawed and resuspended in buffer A (50 mM sodium phosphate + 300 mM NaCl, pH 7.5) supplemented with 2.5 mM imidazole, lysozyme, DNAse, $MgCl_2$, and protease inhibitors, and lysed by cell disruption. Cell debris was removed by centrifugation at 20,000 rpm and 4 °C for 1 h. The collected supernatant was filtered through a 0.22 μm membrane and incubated with 5 mL TALON metal affinity resin (*Clontech*) for 2 h at 4 °C with rolling. Protein-bound resin was washed with buffer A supplemented with 5 mM (wash 1) and 10 mM imidazole (wash 2). Protein was eluted with buffer A supplemented with 150 mM imidazole following 1 h of incubation at 4 °C with rolling. Fractions containing purified protein were further purified by size-exclusion chromatography using a Superdex200 high-performance liquid chromatography column (*Cytiva*), equilibrated with 50 mM phosphate buffer + 150 mM NaCl, pH 7.5. Purified sample was brough to the desired concentration using 10K molecular weight cut off Vivaspin centrifugal filter devices (*Sartorius*).

Native Mass Spectrometry and Ion Mobility Mass Spectrometry CCS Measurements

Mass spectrometry experiments were performed on a modified traveling-wave ion mobility enabled Synapt G2-S (Waters), described previously.[7] The n-ESI tips were pulled in-house from borosilicate capillaries (outer diameter 1.2 mm, inner diameter 0.69 mm, length 10 cm, *Science Products GmbH*) using a laser-based P-2000 micropipette puller (*Sutter Instrument Company*). A positive voltage was applied to the solution *via* a platinum wire (*Goodfellow Cambridge Ltd*). Data were analysed using MassLynx v4.1 (*Waters Corporation*), OriginPro 9.1 (*OriginLab Corporation*), and Microsoft Excel 2010 (*Microsoft*). Native mass spectra were recorded on a modified Synapt G2-S employing gentle source conditions with a capillary voltage between 1.1-1.4 kV, cone voltage of 10 V and all radio frequencies set to zero. $^{TW}CCS_{N2}$ measurements were performed on the same modified mass spectrometer following the standard calibration procedure utilising the Mason-

Schamp equation.[8-10] Measurements were made in nitrogen and spraying conditions were again kept as gentle as possible with an applied capillary voltage range of 1.1 to 1.4 kV, cone voltage maintained at 10 V and a source temperature of 333.13 K.

Table S2. Standard proteins and their ion mobility $^{TW}CCS_{N2}$ values determined by DTIMS. These were used to calibrate the TtCarH TWIMS ions mobility data.

Protein	Mass	Charge State	Literature CCS (Ω_{He}, Å^2)[3]	Literature CCS (Ω_{N2}, Å^2)[5]
Alcohol Dehydrogenase	~148 kDa	26+	6720	7473
		25+	6830	7465
		24+	6940	7447
		23+	6940	7422
BSA	~66 kDa	17+	4040	4523
		16+	4060	4514
		15+	4100	4478
		14+	4090	4425
B-Lactoglobulin (monomer)	~18 kDa	9+	1780	NA
		8+	1690	
		7+	1660	
B-Lactoglobulin (dimer)	~36 kDa	13+	2850	NA
		12+	2900	
		11+	2960	
Concanavalin A	~103 kDa	22+	5550	5930
		21+	5550	5921
		20+	5480	5902

Mass spectrometry titration experiments

WT TtCarH and G192Q variants were buffer exchanged into 250 mM Ammonium Acetate, pH 6.8 using Slide-a-Lyzer Dialysis Cassettes (*ThermoFisher*). The concentration of this new stock solution was then checked by absorption at 280 nm using a DS-11 Spectrophotometer (*Denovix*). The AdoCbl and MeCbl were diluted to the desired concentration in ultrapure water that was obtained from a Milli-Q Advantage A10 ultrapure water filtration system (Merck Millipore, Darmstadt, Germany). Data were acquired for a range of TtCarH:B_{12} ratios, from excess proteins to excess B_{12}. Mixtures at each ratio were made up under red light to avoid photo-activation, vortexed and inserted into the nESI tip, which was then covered and kept in the dark during acquisition. The final monomeric concentration for the mass spectrometry was 12 µM for the apo-protein and an overall concentration of 10 µM for the titration mixtures. Fresh titration mix was prepared for all experimental MS runs and all experiments were performed in triplicate.

Fluorescence stopped-flow measurements

Fluorescence stopped-flow measurements were carried out in a SX20 stopped-flow spectrometer (*Applied Photophysics*), with Pro-Data SX and Pro-Data viewer operating software. All samples were prepared in 150 mM NaCl, 50 mM phosphate, pH 7.5. Protein samples at each working concentration (see Figures 4 and 5) were prepared under white light, whereas the cobalamin samples were prepared under red light and stored in black vials due to their photosensitivity. Stopped flow measurements were performed in a 20 µL quartz cell at ~ 25 ^{o}C, using an excitation wavelength of 280 nm, 2 mm monochromator slit width (entrance and exit), a 320 nm long-pass filter, and 400 V in the fluorescence channel. All measurements were performed under red light to stop unwanted photo-activation.

After loading into the drive syringes, all samples were thermal equilibrated with the water bath for ~ 10 minutes. Fluorescence emission data were acquired following rapid mixing of protein and B_{12} with 1000 data points acquired over the acquisition period. The first three shots were discarded per syringe fill to account for the dead volume, and the kinetic data acquisition repeated for each of the following 5 shots. Each repeat trace was fitted from 0.1 ms to 30 s to an appropriate sum of exponentials using the fitting tool on OriginPro 2020 (*OriginLab Corporation*). Each fitting yielded decay constants that were converted to pseudo first-order rate constants; these were then averaged, and standard deviations and standard errors calculated. Concentration dependences of each rate constants was assessed by plotting the averaged values as a function of both cobalamin and TtCarH concentrations. Second order rates were derived from the gradient of linear fits (Figures 4, 5 and S18, and Table S3).

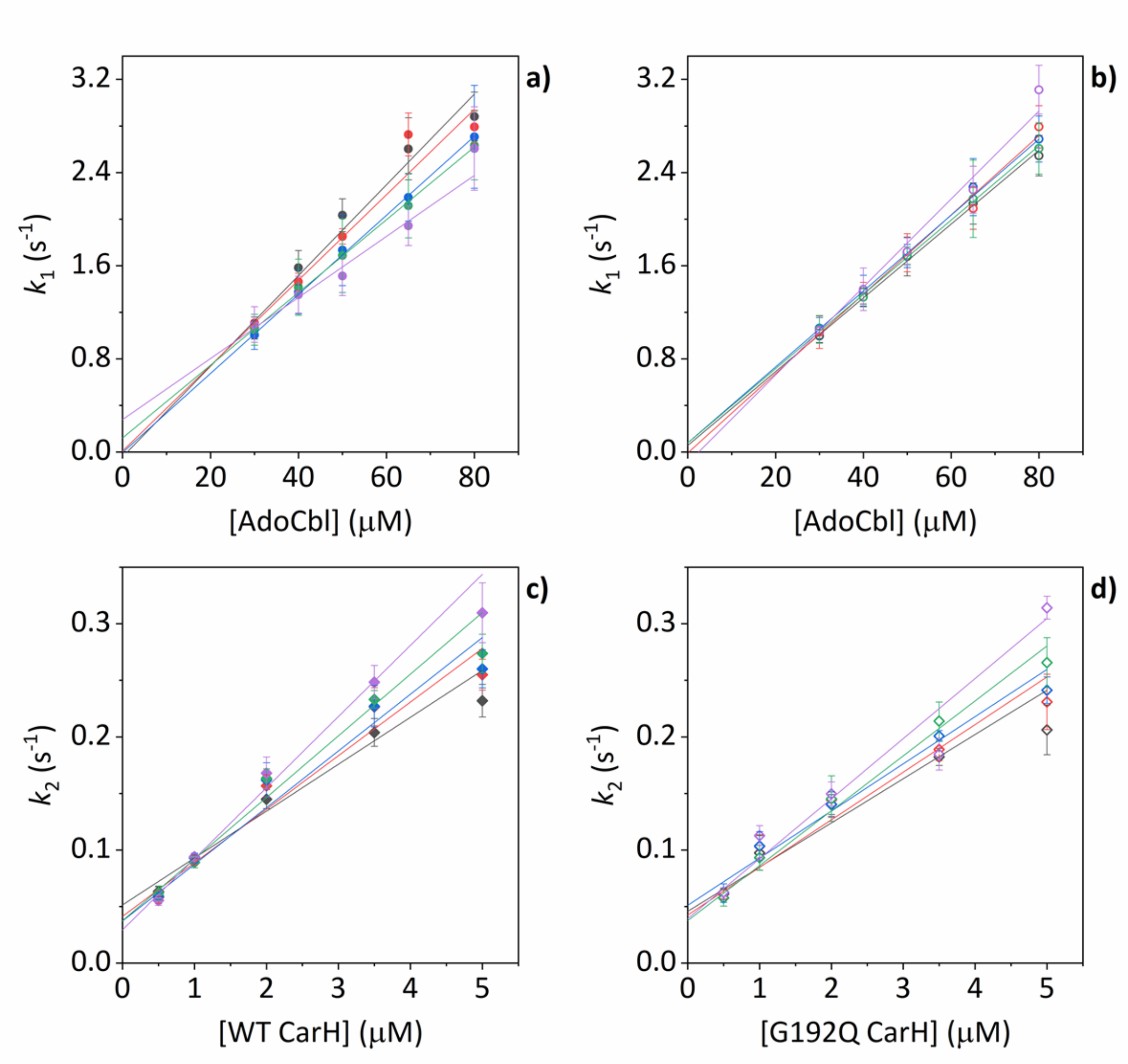

Figure S18. Linear fits of the concentration-dependent kinetic data acquired from the fluorescence stopped-flow experiments. (a-b) Linear fits to the variation of pseudo first order rates as a function of [AdoCbl] for each [TtCarH] for WT **(a)** and G192Q **(b)**. Data points and fit lines are colored according to the [TtCarH]: black, 0.5 μM; red, 1 μM; blue, 2 μM; green, 3.5 μM; purple, 5 μM. **(c-d)** linear fits to the variation of pseudo first order rates as a function of [TtCarH] for WT **(c)** and G192Q **(d)** and each [AdoCbl]. Data points and fits lines are colored according to the [AdoCbl]: black, 30 μM; red, 40 μM; blue, 50 μM; green, 65 μM; purple, 80 μM. The parameters from each fit are in Table S3.

Table S3. Second order rate (slope) and dissociation coefficients (y-intercept) for both B_{12} binding to WT and G192Q TtCarH variants and for their domain assembly. Fitting was conducted in two ways. First, for the [AdoCbl]-dependences, the variation of the pseudo first order rate was fit linearly at each concentration of the two TtCarH variants. Similarly, for the [protein]-dependences, data were fit for each [AdoCbl]. For these fits, the parameters were averaged and errors calculated (highlighted yellow; *c.f.*, Figure S18). Second, concatenated fits were performed (*c.f.*, Figures 4&5), where a single linear fit was made for the [AdoCbl]-dependence to all of the data from every [protein] (separately for WT and G192Q). The same was done for the [protein]-dependences for all [AdoCbl]. This was either conducted including (highlighted orange) or excluding (highlighted grey) the non-pseudo first order data.

		slope (s^{-1} mM^{-1})			y-intercept (s^{-1})			K_D (μM)		
	[WT CarH] | [G192Q CarH] (μM)	value	SE	SD	value	SE	SD	value	SE	SD
k_1 vs [AdoCbl]	0.5	38.94	3.73	-	-0.04244	0.16948	-	-1.0899	-	-
	1	36.67	2.43	-	0.00839	0.07891	-	0.2288	-	-
	2	33.95	0.69	-	-0.00459	0.03064	-	-0.1352	-	-
	3.5	31.23	0.76	-	0.12048	0.03516	-	3.8578	-	-
	5	26.21	2.67	-	0.27840	0.13086	-	10.6219	-	-
	averaged data (calculated)	33.40	2.21	4.95	0.07205	0.05829	0.13035	2.6967	2.1518	4.8116
	concatened fit inc. non-pseudo 1st order data	34.34	1.39	-	0.07675	0.04754	-	2.2350	-	-
	concatened fit exc. non-pseudo 1st order data	36.68	1.43	-	0.00710	0.04791	-	0.1936	-	-
	0.5	31.69	0.80	-	0.05625	0.03312	-	1.7750	-	-
	1	34.07	1.78	-	-0.00818	0.08919	-	-0.2401	-	-
	2	32.60	0.90	-	0.07826	0.04157	-	2.4006	-	-
	3.5	31.91	1.03	-	0.07735	0.04771	-	2.4240	-	-
	5	37.79	2.13	-	-0.09452	0.07211	-	-2.5012	-	-
	averaged data (calculated)	33.61	1.12	2.51	0.02183	0.03308	0.07398	0.7717	0.9520	2.1287
	concatened fit inc. non-pseudo 1st order data	33.38	0.78	-	0.02910	0.03163	-	0.8718	-	-
	concatened fit exc. non-pseudo 1st order data	32.92	0.77	-	0.03096	0.03442	-	0.9405	-	-

		slope (s^{-1} mM^{-1})			y-intercept (s^{-1})			K_D (μM)		
	[AdoCbl] (μM)	value	SE	SD	value	SE	SD	value	SD	SE
k_2 vs [WT CarH]	30	41.46	4.84	-	0.05143	0.00566	-	1.2405	-	-
	40	47.35	5.25	-	0.04132	0.00685	-	0.8727	-	-
	50	50.12	5.29	-	0.03720	0.00587	-	0.7422	-	-
	65	54.47	4.69	-	0.03747	0.00913	-	0.6879	-	-
	80	62.85	4.64	-	0.02936	0.00525	-	0.4671	-	-
	averaged data (calculated)	51.25	3.59	8.02	0.03936	0.00359	0.00803	0.8021	0.1277	0.2855
	concatened fit inc. non-pseudo 1st order data	50.44	2.30	-	0.04055	0.00290	-	0.8039	-	-
	concatened fit exc. non-pseudo 1st order data	55.28	2.60	-	0.03527	0.00347	-	0.6380	-	-
k_2 vs [G192Q CarH]	30	39.08	4.19	-	0.04578	0.00868	-	1.1714	-	-
	40	42.09	4.19	-	0.04251	0.00610	-	1.0100	-	-
	50	41.71	3.41	-	0.05109	0.01047	-	1.2249	-	-
	65	48.55	3.01	-	0.03757	0.00554	-	0.7738	-	-
	80	53.08	4.98	-	0.03922	0.00959	-	0.7389	-	-
	averaged data (calculated)	44.902	2.57	5.75	0.04323	0.00242	0.00541	0.9838	0.0995	0.2225
	concatened fit inc. non-pseudo 1st order data	45.12	1.86	-	0.04211	0.00399	-	0.9333	-	-
	concatened fit exc. non-pseudo 1st order data	46.73	2.40	-	0.04246	0.00580	-	0.9086	-	-

Static fluorescence measurements

Static fluorescence measurements were carried out in a FLS920 spectrofluorometer (*Edinburgh Instruments*), with the F980 spectrometer operating software. Samples were diluted to ~ 0.1 OD at the wavelength of interest with the same buffer used in the stopped-flow measurements. Quartz cuvettes with a fully masked standard exterior size, 10 mm emission aperture window pathlength and 2 mm sample chamber were used. For the photoconversion experiment, a holo-TtCarH sample was illuminated using a high-power light-emitting diode (LED; *Thorlabs*) with λ_{max} = 530 nm.

References

(1) Jost, M.; Fernández-Zapata, J.; Polanco, M. C.; Ortiz-Guerrero, J. M.; Chen, P. Y.-T.; Kang, G.; Padmanabhan, S.; Elías-Arnanz, M.; Drennan, C. L. Structural basis for gene regulation by a B_{12}-dependent photoreceptor. *Nature* **2015,** *526*, 536.

(2) Főrster, T. 10th Spiers Memorial Lecture. Transfer mechanisms of electronic excitation. *Discuss. Faraday Soc.* **1959,** *27* (0), 7.

(3) Eftink, M. R. In *Methods of Biochemical Analysis*, 1991, DOI:https://doi.org/10.1002/9780470110560.ch3 https://doi.org/10.1002/9780470110560.ch3.

(4) Hess, B.; Kutzner, C.; van der Spoel, D.; Lindahl, E. GROMACS 4: Algorithms for Highly Efficient, Load-Balanced, and Scalable Molecular Simulation. *J. Chem. Theory Comput.* **2008,** *4* (3), 435.

(5) Duan, Y.; Wu, C.; Chowdhury, S.; Lee, M. C.; Xiong, G.; Zhang, W.; Yang, R.; Cieplak, P.; Luo, R.; Lee, T.et al. A point-charge force field for molecular mechanics simulations of proteins based on condensed-phase quantum mechanical calculations. *J Comput Chem* **2003,** *24* (16), 1999.

(6) Ortiz-Guerrero, J. M.; Polanco, M. C.; Murillo, F. J.; Padmanabhan, S.; Elías-Arnanz, M. Light-dependent gene regulation by a coenzyme B_{12}-based photoreceptor. *Proc. Natl. Acad. Sci. USA* **2011,** *108* (18), 7565.

(7) Bellina, B.; Brown, J. M.; Ujma, J.; Murray, P.; Giles, K.; Morris, M.; Compagnon, I.; Barran, P. E. UV photodissociation of trapped ions following ion mobility separation in a Q-ToF mass spectrometer. *Analyst* **2014,** *139* (24), 6348.

(8) Ruotolo, B. T.; Benesch, J. L. P.; Sandercock, A. M.; Hyung, S.-J.; Robinson, C. V. Ion mobility–mass spectrometry analysis of large protein complexes. *Nat. Prot.* **2008,** *3* (7), 1139.

(9) Shvartsburg, A. A.; Smith, R. D. Fundamentals of Traveling Wave Ion Mobility Spectrometry. *Anal. Chem.* **2008,** *80* (24), 9689.

(10) France, A. P.; Migas, L. G.; Sinclair, E.; Bellina, B.; Barran, P. E. Using Collision Cross Section Distributions to Assess the Distribution of Collision Cross Section Values. *Anal. Chem.* **2020,** *92* (6), 4340.
